# Proposed Lyme Disease Guidelines and Psychiatric Illnesses

**DOI:** 10.3390/healthcare7030105

**Published:** 2019-09-09

**Authors:** Robert C. Bransfield, Michael J. Cook, Douglas R. Bransfield

**Affiliations:** 1 Department of Psychiatry, Rutgers-Robert Wood Johnson Medical School, Piscataway, NJ 08854, USA; 2 Independent Researcher, Dorset BH23 5BN, UK; mcook98@msn.com; 3 General Counsel Red Paladin, Piscataway, NJ 08854, USA; Dbransfield@redpaladin.com

**Keywords:** Lyme disease, guidelines, standards, psychiatric, mental illnesses, Infectious Disease Society of America (IDSA), American Academy of Neurology (AAN), American Academy of Rheumatology (AAR), disclaimer

## Abstract

The Infectious Disease Society of America, American Academy of Neurology, and American Academy of Rheumatology jointly proposed Lyme disease guidelines. Four areas most relevant to psychiatry were reviewed—the disclaimer, laboratory testing, and adult and pediatric psychiatric sections. The disclaimer and the manner in which these guidelines are implemented are insufficient to remove the authors and sponsoring organizations from liability for harm caused by these guidelines. The guidelines and supporting citations place improper credibility upon surveillance case definition rather than clinical diagnosis criteria. The guidelines fail to address the clear causal association between Lyme disease and psychiatric illnesses, suicide, violence, developmental disabilities and substance abuse despite significant supporting evidence. If these guidelines are published without very major revisions, and if the sponsoring medical societies attempt to enforce these guidelines as a standard of care, it will directly contribute to increasing a national and global epidemic of psychiatric illnesses, suicide, violence, substance abuse and developmental disabilities and the associated economic and non-economic societal burdens. The guideline flaws could be improved with a more appropriate disclaimer, an evidence-based rather than an evidence-biased approach, more accurate diagnostic criteria, and recognition of the direct and serious causal association between Lyme disease and psychiatric illnesses.

## 1. Introduction

The Infectious Disease Society of America (IDSA), in combination with the American Academy of Neurology (AAN) and the American Academy of Rheumatology (AAR) draft guidelines were recently temporarily released for public comment in 2019 [1]. The current draft contains a section on adult patients with psychiatric illness and children with developmental, behavioral or psychiatric disorders. Section XII, page 38, line 881 states “Should adult patients with psychiatric illnesses be tested for Lyme disease?” Section XIII, page 39, line 897 states “Should children with developmental, behavioral or psychiatric disorders be tested for Lyme 897 disease?” [1]

The United States is experiencing an epidemic of increasing mental illness, suicide, violence, substance abuse and developmental disabilities in children, and many are questioning why this is occurring [2]. At the same time, there is an increasing epidemic of Lyme and other tick-borne diseases. There is also increasing recognition that infections and chronic tick-borne infections are contributing significantly to a burden of mental illness, suicide, violence, developmental disabilities in children and substance abuse [3]. A review of the causal association between Lyme disease and psychiatric illness has recently been performed [3]. Psychiatric illnesses caused by Lyme disease, include developmental disorders, autism spectrum disorders, schizoaffective disorders, bipolar disorder, depression, anxiety disorders (panic disorder, social anxiety disorder, generalized anxiety disorder, posttraumatic stress disorder, intrusive symptoms), eating disorders, decreased libido, sleep disorders, addiction, opioid addiction, cognitive impairments, dementia, suicide, violence, anhedonia, depersonalization, dissociative episodes, derealization and other impairments [3]. These impairments result in diminished quality of life, lost productivity, disability, caregiver burden, violence, and suicide from untreated and inadequately treated Lyme disease [3]. Lyme disease causes an estimated 1200 suicides, 31,000 suicide attempts and 15,000 self-harm events in the United States per year [4]. Other psychiatric mortality from Lyme disease includes drug overdoses, accidents from cognitive impairments and homicide [3]. Clearly, inadequate diagnosis and treatment of Lyme disease has resulted in significant psychiatric morbidity and mortality. This is quite relevant when making risk vs. benefit diagnostic and treatment decisions regarding Lyme disease.

Many within the United States and globally follow IDSA guidelines, assuming that they are scientifically accurate, and errors in these guidelines could have very serious adverse effects. Lyme disease guidelines would be more trustworthy if they sufficiently address a major cause of morbidity and mortality—psychiatric illness. Conversely, if the proposed guidelines fail to address psychiatric illness, the dissemination of inaccurate guidelines combined with efforts to impose such guidelines as standards of care can have serious liability implications.

The 2006 IDSA guidelines failed to recognize that psychiatric disorders and developmental disabilities are directly caused by Lyme disease, were widely criticized and became a focal point of a major lawsuit [5,6,7,8,9,10,11,12,13,14]. If the new guidelines instead recognize the significance and seriousness of psychiatric illnesses caused by Lyme disease and incorporate this research into their guidelines, it would have a major impact upon the risks associated with the under diagnosis of Lyme disease. Such an inclusion would also significantly impact risk vs. benefit treatment recommendations throughout the guidelines and would be an improvement over the 2006 IDSA Lyme disease guidelines.

The Method section of the guidelines state “they are informed by a systematic review of evidence.” The current draft contains a section on adult patients with psychiatric illness and children with developmental, behavioral or psychiatric disorders. These sections contain a total of only four citations [1]. A systematic review of the psychiatric evidence would be most helpful in assessing the validity of the proposed Lyme disease guidelines.

## 2. Materials and Methods

The sections most directly relevant to psychiatry in the 100-page document were selected for review. Those sections include the disclaimer (Page 1, Line 4 footnote), laboratory testing (Line 224–85), the testing of adults with psychiatric illness (Page 38, Line 881–96) and the testing of children with developmental, behavioral and psychiatric disorders (Page 39, Line 897–4). PubMed, Google Scholar and authors’ archives were searched to evaluate the scientific accuracy of statements made in the proposed guidelines. These sections were then compared to the medical literature and analyzed to evaluate whether they were evidence based or evidence biased, and conclusions were drawn from this analysis.

Since the stated method in the guidelines was “a systematic review of evidence,” a systematic review of evidence between Lyme disease and psychiatric illness was conducted with limited use of the Preferred Reporting Items for Systematic Reviews and Meta-Analysis (PRISMA) method and compared with the results of the psychiatric references cited in the proposed guidelines. A full PRISMA systemic review and meta-analysis did not appear needed to assess the validity of the systematic review since only four articles were cited in the proposed guidelines. An electronic search of the National Center for Biotechnology Information of the United States National Institutes of Health, National Library of Medicine PubMed Central (PubMed) was conducted with the following terms: “Lyme disease causing psychiatric illness, Lyme disease causing mental illness, Lyme disease causing developmental disorders children, Lyme disease causing behavioral disorders children, and Lyme disease causing psychiatric disorders children”. Duplicate searches were performed by both author RB and MC. The electronic searches chosen to be included in the data analysis were “Lyme disease causing psychiatric illness” in PubMed, “Lyme disease causing psychiatric illness” in Google Scholar, “Lyme disease causing developmental disorders children” in PubMed, and “Lyme disease psychiatric disorders children” in PubMed. The plan was to eliminate any records from the search if there was not consensus between both authors. The results of this search were combined with current lists of articles demonstrating a causal association between Lyme disease and psychiatric illness in the first author’s archives. Articles were separated into different groups. These groups included articles that addressed a causal association between Lyme disease and psychiatric symptoms, pathophysiology, both psychiatric symptoms and pathophysiology, a causal association between Lyme disease pediatric psychiatric symptoms, pediatric psychiatric pathophysiology, pediatric psychiatric symptoms and pathophysiology, and psychiatric symptoms associated with other tick-borne diseases. The results were then compared to the list of citations used in the proposed guidelines to evaluate the thoroughness of the systematic review performed in the guideline draft. An archive of the articles reviewed is made available. The summary of the findings was sent to the website for the IDSA, AAN and AAR guidelines in their required format within their time requirement and was also submitted for peer review.

## 3. Results

### 3.1. The Disclaimer, Guidelines or an Imposed Standard?

The disclaimer is more extensive than in other IDSA guidelines. It recognizes that guidelines are unable to allow for individual patient variations, are not intended to replace physician judgment and adherence to the guidelines is voluntary. In addition, they make no warranty of accuracy or reliability. They hold themselves harmless from any losses from reliance upon the guidelines. Although the disclaimer states that it holds the authors and sponsoring organizations as harmless, the disclaimer does not state that the guidelines cannot be used to attempt to establish a standard of care in quality assurance or legal proceedings. This is very different from the Centers for Disease Control and Prevention (CDC) disclaimer regarding Lyme disease surveillance, which states “Surveillance case definitions establish uniform criteria for disease reporting and should not be used as the sole criteria for establishing clinical diagnosis, determining the standard of care necessary for a particular patient, setting guidelines for quality assurance, or providing standards for reimbursement.” The CDC recognizes their criteria are not synonymous with standards of care. The failure to state that their guidelines cannot be used to establish a standard of care is compatible with policy implemented by the IDSA. There have been multiple testimonies by IDSA physicians against physicians who do not follow the IDSA Lyme disease guidelines as a standard of care. This approach has been and is still being used in various legal proceedings, including state board and malpractice cases. This issue is of such a significance that several states passed legislation protecting physicians from such actions. The seriousness of this problem came to the attention of the United States 2018 Tick-Borne Disease Working Group and this was discussed in the United States 2018 Tick-Borne Disease Working Group Report to Congress, which stated:

“7.6: Protect the rights of licensed and qualified clinicians to use individual clinical judgment, as well as recognized guidelines, to diagnose and treat patients in accordance with the needs and goals of each individual patient.

In endemic states, many providers who treat persistent Lyme disease and other tick-borne diseases with long-term antibiotics risk their livelihoods and reputations to do so. Other clinicians accuse them of compromising the health of the patient, and state medical boards prosecute them for operating outside the IDSA guidelines. These prosecutions have led doctors to feel hesitant about handling chronic or recurrent cases, forcing patients in some instances to seek treatment beyond their home states.

The IDSA guidelines for treating Lyme disease contain a footnote with the following statements: ‘It is important to realize that guidelines cannot always account for individual variation among patients [5].’ They are not intended to supplant physician judgment with respect to particular patients or special clinical situations. The Infectious Diseases Society of America considers adherence to these guidelines to be voluntary, with the ultimate determination regarding their application to be made by the physician in the light of each patient’s individual circumstances’” [5].

Despite the footnote, state licensing boards subject medical providers to disciplinary action and fines for choosing to determine the direction of their patients’ treatments based on their clinical judgment, other recognized diagnostic and treatment guidelines, individual circumstances, and previous treatment responses. Therefore, it falls on each state to produce legislation or policy solutions to promote public awareness and protection for patients and providers. Advocates have successfully achieved those solutions in several states to date.” [15].

“Relay Information as a Neutral Knowledge Broker: The Federal Government cannot endorse one set of treatment guidelines over another, yet it can clarify the intended purpose of its surveillance criteria and recognize all third-party guidelines that meet pre-defined standards and criteria” [15].

Another section in the report to Congress addressed the unreliability of the surveillance case definition. Both the IDSA 2006 and the proposed 2020 guidelines incorrectly use the highly restrictive surveillance case definition as a diagnostic criterion. The CDC recognizes that the surveillance case definition only captures 10% of the actual cases of Lyme disease [16].

“Engage Diverse Stakeholders—Update the CSTE Surveillance Case Definition with 21st-Century Evidence: Data collection and scientific understanding have evolved since the 1994 Dearborn conference (see chapter 5 on diagnosis), yet Lyme disease diagnostics and surveillance criteria remain unchanged. It is time to revisit the Dearborn conference outcomes by convening a meeting of all relevant stakeholders—including government scientists, academic researchers, industry leaders, treating clinicians, patients, family members, and advocates—to review the evidence and interpretive criteria using all the newest diagnostic methodologies, techniques, technologies, and emerging science. Diverse stakeholders, the Working Group, CDC, National Institutes of Health, Federal Drug Administration, and Council of State and Territorial Epidemiologists could examine the science and “real world evidence”—including clinician data and patient registries—to co-create new outcomes and criteria that supersede outdated ones” [15].

In response to the report to Congress, a letter from the president of the IDSA describes the official position of the IDSA, claiming their opinions are “evidence based” and valid and anyone following guidelines disagreeing with their opinions, such as the International Lyme and Associated Diseases Society, are not what they call “evidence based” and they should be exposed to “redress” and “censure.” The letter goes on to state: 

“IDSA has grave concerns about the content in the Access to Care chapter. If the recommendations were implemented as written, they would essentially remove any accountability for physicians providing unproven treatments to patients who may or may not have Lyme disease. These treatments can be harmful, and the recommendations in this chapter would remove patients’ opportunity for redress and prohibit state medical boards from censuring these doctors or preventing them from harming additional patients.

IDSA supports patient access to evidence-based, medically-appropriate diagnosis and treatment of Lyme disease including persistent symptoms that are safe and effective. The recommendations and policies outlined in this chapter would subject patients to faulty diagnostic procedures and dangerous, unproven treatments. We also oppose recommendations or laws designed to protect clinicians who provide harmful treatments. In addition, we oppose any attempts by the Working Group to undermine widely accepted medical guidelines for the treatment of Lyme disease that are rooted in scientific evidence or to promote clinical guidelines that are not evidence-based. We are apprehensive about the potential impact of the recommendation to provide protections for doctors who follow ‘recognized guidelines.’ The term is exceedingly broad and could easily be applied to guideline recommendations that lack enough evidence or are based mainly on patient preference such as the International Lyme and Associated Diseases Society guidelines that give physicians broad latitude regardless of documented efficacy or safety… This is a highly significant oversight and defect. Broad protection for physicians who subject patients to substandard or even dangerous therapies will likely increase the number of patients who are harmed” [17].

### 3.2. Laboratory Testing in the Proposed Guidelines

A critical flaw throughout the entire proposed disease guidelines and the supporting references is the scientifically unfounded use of surveillance case definition as clinical diagnostic criteria and the incorrect assumption that a patient who does not meet Lyme disease surveillance case definition does not meet Lyme disease clinical diagnostic criteria and does not have Lyme disease.

“This surveillance case definition was developed for national reporting of Lyme disease; it is not intended to be used in clinical diagnosis. Surveillance case definitions establish uniform criteria for disease reporting and should not be used as the sole criteria for establishing clinical diagnosis, determining the standard of care necessary for a particular patient, setting guidelines for quality assurance, or providing standards for reimbursement” [18].

A requirement for diagnosing Lyme disease in these proposed guidelines is a positive two-tiered test. That is a positive (or equivocal) enzyme-linked immunosorbent assay (ELISA) test followed by a positive immunoblot. The method was defined for use by the United States Centers for Disease and Prevention (CDC) to standardize surveillance at the 1994 Dearborn meeting and was recommended for clinical diagnosis at one of the workshops.

The most important parameters of this and other biomarker tests is the sensitivity, the probability that a positive sample will test positive, and the specificity, the probability that a negative sample will test negative. If either of these parameters differs from 100%, then there will be consequences.

Sensitivity less than 100% will result in true cases being missed and the patients going untreated with the prospect of serious and long-standing illness. Specificity less than 100% will result in false positive tests which could result in unnecessary treatment. However, the very low risk associated with this is easily demonstrated by the lack of severe adverse events related to billions of doses of antibiotics used each year.

The proposed guidelines state (Line 231–2) “Serum antibody tests should be performed using a two-tiered testing protocol employing clinically validated assays”. This is supported by their reference to the 1995 CDC Lyme disease case definition, [18] and the 2008 Prospective Study of Serologic Tests for Lyme Disease by Steere et al [19].

References that Demonstrate the Low Sensitivity of the Two-Tiered Test:

1. Quote: “For the diagnosis of Lyme disease, the 2-tier serologic testing protocol for Lyme disease has a number of shortcomings including low sensitivity in early disease”. [20] (Wormser GP et al., 2012). This is by Wormser, Aguaro-Rosenfeld and Steere, all co-authors of the proposed 2020 IDSA guidelines. The paper gives specific sensitivities which not only show the method has low sensitivity for early disease but also in the general case where sensitivity is 50.6%. That is 50% of all patients with Lyme disease will not be treated for the disease based on the guidelines.
2. Quote: “The sensitivity of C6 testing (69.5%) was greater than that of 2-tier testing (38.9%).” (Wormser GP et al., 2008). This is again by Wormser and Aguero-Rosenfeld [21].
3. For early stage disease, when it is most important to diagnose the disease for successful treatment, Branda, Lantos, Strle and Steere, who are all authors of the new guidelines, stated: “Sensitivity of the various MTTT (Modified Two-Tier Test) protocols in patients with acute erythema migrans ranged from 36% (95% confidence interval [CI], 25–50%) to 54% (95% CI, 42–67%), compared with 25% (95% CI, 16–38%) using the conventional protocol.” (Branda JA. et al. 2017) [22].
4. In a paper published by Branda with co-authors Aguero-Rosenfeld, Wormser and Steere, the sensitivity of the two tier test for acute cases was 30% and for convalescent cases was 59%, and it was recommended that the test be replaced by a more sensitive one. (J. A. Branda et al., 2010) Of note is that four of the study authors are authors of the proposed 2020 IDSA guideline [23].
5. A report based on the work of Leeflang et al. (2016) [24] indicated that data from 15 studies of the sensitivity of the two-tier test varied from 4% to 50%. A quotation from the report was: “Goossens et al. (2000) evaluated two-tiered tests in early and in late LB. The results were comparable to those for the study with healthy controls, except that there was more variation in specificity. None of the evaluations revealed sensitivity above 50% (one was 50%).” (Zeller and Van Bortel, 2016) [25].
6. A statistical analysis of the two-tier test demonstrated that it generated up to 500 times more false negative results than HIV testing. (Cook and Puri, 2017) [26].
7. A meta-analysis of commercial test kits used with the two-tier protocol identified seven studies. The mean sensitivity for all stages of disease was 53.7% and ranged from 38.9% (Wormser et. al. 2008) to 67.5% (Bacon et al. 2003). (Cook and Puri, 2016) [21,27,28].

### 3.3. Testing Adult Patients with Psychiatric Illness for Lyme Disease

None of the guideline panel members were psychiatrists. The proposed guidelines stated “XII Should adult patients with psychiatric illnesses be tested for Lyme disease?” (Line 881) The question may be irrelevant since they are only recommending surveillance criteria testing. Psychiatric illness is a late stage manifestation of Lyme disease. Surveillance testing criteria has never been standardized for late stage disease. A more appropriate question is whether to perform an adequate screening and total clinical assessment on psychiatric patients who might have Lyme disease. The consideration of Lyme disease in the assessment of psychiatric patients is already recommended in the American Psychiatric Association Practice Guidelines for the Evaluation of Adults, Third Edition [29].

The proposed guidelines recommend against testing for Lyme disease in patients with psychiatric illness. Although they state there is low-quality evidence for this recommendation, they state it is a strong recommendation. They cite four studies to support their position. To support this opinion, they make the following statement “No studies suggest a convincing causal association between Lyme disease and any *specific* psychiatric conditions.” The relevant issue is not *specific* psychiatric conditions, but instead *psychiatric illness* as stated in their question. To support their claim, they gave only four references, which are described below.

The Hájek et al. study recognized a potential association between *Borrelia burgdorferi* infection and psychiatric morbidity [30]. This was the second study Hájek et al. performed. In the first study, Hájek et al. concluded “These findings support the hypothesis that there is an association between *Borrelia burgdorferi* infection and psychiatric morbidity.” [31] In both studies, it was demonstrated that *Borrelia burgdorferi* infections were not associated with a single psychiatric illness, but instead were associated with multiple psychiatric illnesses [30,31].

The Koola et al. study demonstrated that some, but not all, cases of schizophrenia may be caused by Lyme disease. The conclusion was “In conclusion, current data illustrates the important role that healthcare practitioners have in emphasizing Lyme disease as a potential health concern, as well as the opportunity to promote strategies to prevent contraction. Clinicians working in the endemic, high-risk areas should consider Lyme disease in the differential diagnosis of any atypical psychiatric presentation. Further research is warranted to investigate the differential diagnosis of Lyme disease and schizophrenia and examine the mechanisms and treatment of Lyme disease in mental illness” [32].

The 1997 Nadelman et al. study and the Zomer study, which only looked at depression and used CDC surveillance criteria rather than clinical diagnostic criteria, were the only two negative studies that failed to prove a causal association [33,34]. In contrast, there are well over 300 citations demonstrating a causal association between Lyme disease and psychiatric illness and another seventy demonstrating a causal association between Lyme disease and Alzheimer’s disease [35]. The more recent articles published in the past two years have not been included in the list of 300 citations [35].

Contrary to the statement made in the proposed guideline, several studies have found a causal association between Lyme disease and specific psychiatric illnesses [3,4,36]. Twenty different groups of patients with Lyme disease have been studied to evaluate for the presence of specific psychiatric illnesses. There was a significant presence of psychiatric illnesses and psychiatric comorbidity in these patients post-infection. When documented, there was a low prevalence of mental illnesses in these patients before infection. Post infection, psychiatric illnesses included depression/dysphoria: 37%, 37%, 50%, 51%, 64%, 70%, 76%, 80%, 97%, 98%, and 100%; bipolar disorder: 5%, 10%, 19%, 20%, 21%, and 28%; panic disorder: 35%, 50%, 54%, 80%, and 82%; obsessive compulsive disorder: 32%, 42%, 44%, 51%, and 84%; social anxiety disorder: 20%, 55%, 65%, 66%, 68%, and 70%; generalized anxiety disorder: 50%, 65%, 70%, 86%, and 90%; posttraumatic stress disorder: 15%, 15%, 24%, 30%, and 36%; depersonalization: 40%, 52%, 55%, 71%, and 76%; derealization: 24%, 32%, and 37%; paranoia: 10%, 25%, 36%, 62%, 76%, and 88%; anhedonia: 56%, 59%, 71%, 72%, and 85%; suicidal: 20%, 43%, 46%, 63%, 72%, and 98%; substance abuse: 5%, 10%, 10%, 28%, and 33%; executive functioning impairments: 98%; reading comprehension impairments: 79%; auditory comprehension impairments: 73%; dysfluent speech: 46%, 75%, 79%, and 82%; working memory impairments: 98%; recent memory impairments: 94%; attention span impairments: 77% and 77%; sensory hypersensitivity: 86%, hypersensitivity to sound: 58% and 88% and hypersensitivity to light: 74% [3,4,36].

The proposed guidelines state that “there is no controlled prospective evidence that treatment for Lyme disease is effective for any specific psychiatric disease.” There never will be any controlled prospective studies on patients with neuropsychiatric manifestations from Lyme disease since withholding treatment in such a study would invariably be a repeat of the Tuskegee trial and would be highly unethical.

The proposed guidelines state that “While studies have found evidence of exposure to tick-borne infections in some psychiatric patients, there has not been clear etiologic evidence linking the psychiatric disease to infection.” Two studies clearly demonstrate the prevalence of mental illness pre-infection compared to post-infection [4,36]. In addition, studies have demonstrated the psychoimmunology that explains how Lyme disease can result in psychiatric illness [37,38,39,40,41,42].

### 3.4. Testing Children with Developmental, Behavioral or Psychiatric Disorders for Lyme Disease

None of the panel members were child psychiatrists. The proposed IDSA guidelines addressed the following question “XIII. (Line 897) Should children with developmental, behavioral or psychiatric disorders be tested for Lyme disease?” Although they recognized the Greenberg study suggesting a causal association between Lyme disease and childhood bipolar illness in their Supplement Materials (Page 122), it was not included in the guidelines [43]. They recommend “In children presenting with developmental, behavioral or psychiatric disorders, we suggest against routinely testing for Lyme disease *(weak recommendation, low-quality evidence).”* They state “There are no data to support a causal relationship between tick-borne infections and childhood developmental delay or behavioral disorders (such as attention deficit-hyperactivity disorder, autistic spectrum disorders, Pediatric Autoimmune Neuropsychiatric Disorders Associated with Streptococcal Infections (PANDAS), learning disabilities, or psychiatric disorders.” They state “As with many acute medical illnesses, Lyme disease could worsen behavioral or psychiatric symptoms in children who have psychiatric disorders. As with many acute medical illnesses, Lyme disease could worsen behavioral or psychiatric symptoms in children who are predisposed to them. There are no data that associate Lyme disease and developmental or behavioral disorders.” No references are cited in this section. A review of the medical literature revealed multiple articles demonstrating a causal relationship between Lyme disease and childhood cognitive impairments, autism spectrum disorder, PANDAS, learning disabilities and childhood disorders [28,29,33,44,45,46,47,48,49,50,51,52,53,54,55,56,57,58,59,60,61,62,63,64,65,66].

### 3.5. Systematic Review of Lyme Disease Causing Psychiatric Illness

The PubMed electronic search of “Lyme disease psychiatric illness” by RB resulted in 1054 citations on 23-8-2019 and 28-8-2019. The PubMed electronic search of “Lyme disease psychiatric illness” by MC on 28-8-2019 and 28-8-2019 resulted in 1054 citations The PubMed search of “Lyme disease causing psychiatric illness” by RB resulted in 384 citations on 24-8-2019. The PubMed electronic search of “Lyme disease causing Psychiatric Illness” by MC on 28-8-2019 resulted in 384 citations. The PubMed electronic search of “Lyme disease causing mental illness” by RB on 24-8-2019 resulted in 413 citations. The PubMed search of “Lyme disease causing mental illness” by MC on 28-8-2019 resulted in 413 citations. The Google Scholar search of “Lyme disease psychiatric illness” by RB on 28-8-2019 resulted in 21,700 citations. 

The Google Scholar search of “Lyme disease psychiatric illness” by RB on 28-8-2019 resulted in 21,700 citations. The Google Scholar search of “Lyme disease psychiatric illness” by MC on 28-8-2019 resulted in 21,700 citations. The PubMed search of “Lyme disease causing developmental disorders children” by RB on 24-8-2019 resulted in 134 citations. The PubMed search of “Lyme disease causing developmental disorders children” by MC on 28-8-2019 resulted in 134 citations. The search of “Lyme disease causing behavioral disorders children” by RB on 24-8-2019 resulted in 268 citations. The PubMed search of “Lyme disease causing behavioral disorders children” by MC on 28-8-2019 resulted in 268 citations. The PubMed search of “Lyme disease psychiatric disorders children” by RB on 24-8-2019 resulted in 267 citations. The PubMed search of Lyme disease psychiatric disorders children by MC on 28-8-2019 resulted in 267 citations. The first 400 citations from the Google Scholar search were added to the other three searches which resulted in 1185 citations. There was consensus between both authors in the electronic searches. 

**Figure 1:**
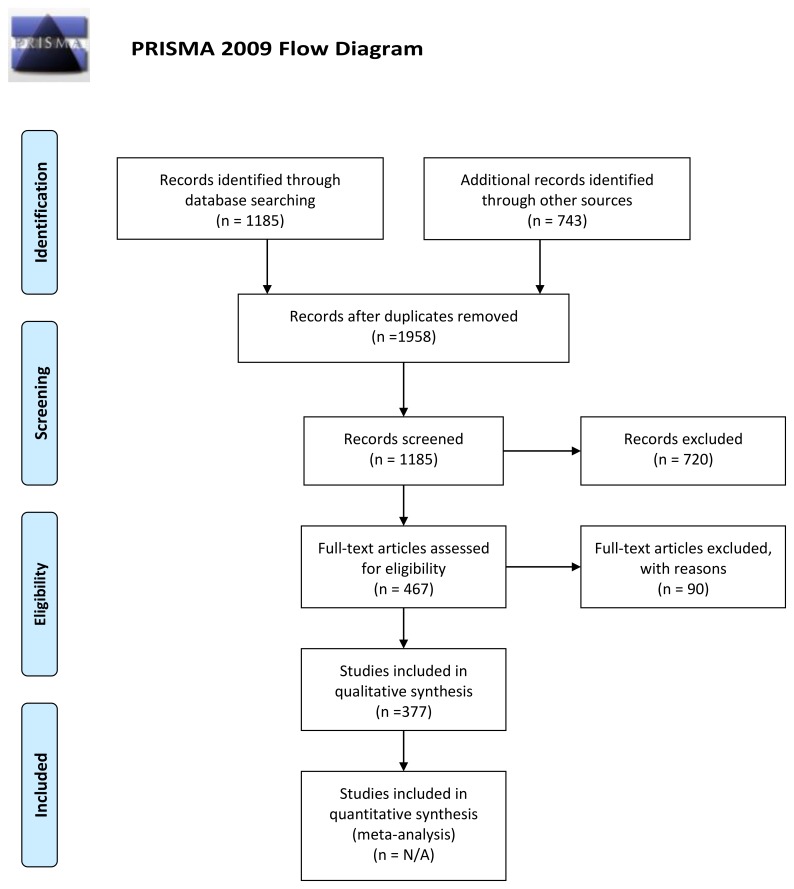
Lyme disease causing psychiatric illness [70].

The list of articles demonstrating a causal association between Lyme disease and psychiatric illness on the International Lyme and Associated Diseases (ILADS) website contained 377 citations, 304 were psychiatric illness and 73 were dementia. [35] The list of articles demonstrating a causal association between Lyme disease and psychiatric illness in the first author’s personal archives contained 389 citations. These two lists were combined for a total of 773 citations. Combining this with the PubMed search resulted in 1958 citations. There were 1185 records after duplications were removed. There were 720 records excluded that did not demonstrate a causal association between Lyme disease and psychiatric symptoms. There were 467 articles assessed for eligibility and 90 were excluded. In this group, 14 addressed other tick-borne diseases, one was an animal behavior study, 73 specifically focused on dementia, and there were two in which the results did not match the conclusion. This resulted in 377 articles demonstrating a causal association between Lyme disease and psychiatric symptoms (Figure 1). Most of these articles had been recently reviewed by the first author when writing a recent review article [3]. Within these 377 articles, there were 258 that addressed psychiatric symptoms caused by Lyme disease, 41 that addressed pathophysiology, and 46 that addressed psychiatric symptoms and pathophysiology. Eleven addressed pediatric symptoms, three addressed pediatric pathophysiology of psychiatric symptoms and eight addressed both pediatric symptoms and pathophysiology. Supplemental material, “Peer-Reviewed Evidence of Lyme/Tick-Borne Diseases Causing Psychiatric Symptoms” is included with this article for reference. 

Two studies were found in the electronic search that deserved further comment. They were two additional negative studies that were not referenced in the proposed guidelines; however, in both studies, the results did not match the conclusions [67,68].

One study assessed mental health scores of Lyme disease patients 11–20 years post infection and concluded that these scores were like those of the general population [67]. The study had multiple flaws. These flaws included a failure to perform psychiatric assessments; the inclusion of only 35% of the initial study group, which suggests a selection bias; the inclusion of only subjects who were effectively diagnosed and treated early; the use of assessment scales insufficient to adequately evaluate the cognitive and psychiatric impairments seen in Lyme disease; a failure to differentiate between statistical vs. clinical significance; and a research design that was not powered a priori to detect differences in functional outcomes [69]. 

In the other study, the authors identified a group that was labeled as chronic multi-symptom illness or post Lyme disease syndrome [68]. These were patients who met CDC surveillance criteria for Lyme disease and, in most cases, the two-tiered protocol for laboratory tests and had received what the authors considered adequate prior antibiotic treatment defined by guidelines from the Infectious Diseases Society of America, but these patients continued to report persistent psychiatric symptoms ascribed to Lyme disease [5,68]. Their psychiatric symptoms included any clinical psychiatric disorder, 48.4%; current depression, 26.3%; past depression, 3.2%; depression/dysthymia, 16.1%; anxiety disorder, 29%; panic disorder, 12.9%; and generalized anxiety disorder, 25.8% [68]. Their study failed to assess a baseline of the mental health of these patients prior to infection. Therefore, a significant amount of the psychiatric comorbidity described in their study may have been acquired post-infection. However, they concluded that “psychiatric comorbidity and other psychological factors are prominent in the presentation and outcome of some patients who inaccurately ascribe longstanding symptoms to chronic Lyme disease” [3,69].

The review conducted by the authors resulted in 377 records supporting an association between Lyme disease and psychiatric illness. By contrast, the “systematic review” of the proposed guidelines resulted in only four articles. These articles appeared to be limited to epidemiological studies and only two of these articles failed to show a causal association between Lyme disease and psychiatric illness [1]. As a result, this gives a clear appearance of a bias related to the selective reporting of outcomes, which makes this part of the guidelines evidence biased, rather than evidence based.

## 4. Conclusions

The IDSA/AAN/AAR Lyme guidelines disclaimer is more extensive than the disclaimer used in other IDSA guidelines, but the wording of the disclaimer and the way the guidelines are implemented are insufficient to remove the authors and sponsoring organizations from liability for harm caused by these guidelines. The guidelines, as currently proposed, place improper credibility upon surveillance definition not intended for diagnosis rather than clinical diagnosis criteria. In addition, the guidelines are highly dependent upon research and journal articles that make this same mistake. The proposed guidelines fail to recognize the clear causal association between Lyme disease and psychiatric illnesses in children and adults that may include suicide, violence, substance abuse and developmental disabilities. Suicide is a major cause of mortality in patients with Lyme disease [4]. These errors result in this part of the guidelines being evidence biased rather than evidence based. If the causal association between Lyme disease and psychiatric illnesses is not recognized in the guidelines, it would have serious adverse consequences when making treatment risk vs. benefit decisions. 

If these guidelines are published without very major revisions, and if the sponsoring medical societies attempt to enforce these guidelines as a standard of care, this will directly contribute to a national and global epidemic of psychiatric illnesses, suicide, violence, substance abuse and developmental disabilities in children and adults that could otherwise be reduced if these guidelines were instead truly evidence based and more scientifically accurate.

## Supplementary Materials

The following are available online at https://www.mdpi.com/2227-9032/7/3/105/s1, Peer-Reviewed Evidence of Lyme/Tick-Borne Diseases Causing Psychiatric Symptoms.
