# Supplementary material for: Proposed Lyme Disease Guidelines and Psychiatric Illnesses
Source: Healthcare (Basel). 2019 Sep 9;7(3):105. doi: 10.3390/healthcare7030105 (PMC6787753; doi:10.3390/healthcare7030105)
Supplement: Supplementary file 1 [file healthcare-07-00105-s001.pdf]

Peer-Reviewed Evidence of Lyme/Tick-Borne Diseases Causing Psychiatric Symptoms

The following is a list of peer-reviewed articles that support the evidence of Lyme and other tick-borne diseases causing neuropsychiatric illness. It is organized into two different categories— neuropsychiatric symptoms and dementia.

Lyme/Tick-Borne Diseases and Neuropsychiatric Symptoms

1. Aalto A, Sjowall J, Davidsson L, Forsberg P, Smedby O. Brain magnetic resonance imaging does not contribute to the diagnosis of chronic neuroborreliosis. Acta Radiol 2007; 48: 755-762. [white matter hyperintensities or basal ganglia lesions].
2. Ackermann R, Rehse-Kupper B, Gollmer E, Schmidt R.Chronic neurologic manifestations of erythema migrans borreliosis. Ann N Y Acad Sci. 1988;539:16-23.
3. Adams WV, Rose CD, Eppes SC, Klein JD. Long-term cognitive effects of Lyme disease in children. Appl Neuropsychol 1999;6(1):39-45
4. Alaedini A, Latov N Antibodies against OspA epitopes of Borrelia burgdorferi cross- react with neural tissue. J Neuroimmunol. 2005 Feb;159(1-2):192-5. Epub 2004 Nov 26.
5. Almeida OP, Lautenschlager NT. Dementia associated with infectious diseases. Int Psychogeriatr. 2005;17 Suppl 1:S65- 77. Review.
6. Amsterdam JD, O'Reardon JP. Treatment-Resistant Depression: Progress and Limitations. Psychiatric Annals. 1998;28(11):633
7. APA Work Group on Psychiatric Evaluation. The American Psychiatric Association Psychiatric Guidelines for the Psychiatric Assessment of Adults Third Edition.
8. Asadipooya K, Dehghanian A, Omrani GH, Abbasi F. Short-course treatment in neurobrucellosis: a study in Iran. Neurol India. 2011 Jan-Feb;59(1):101-3.
9. Asch ES, Bujak DI, Weiss M, Peterson MG, Weinstein A. Lyme disease: an infectious and postinfectious syndrome. J Rheumatol. 1994 Mar;21(3):454-61
10. Asha'ari ZA, Mat Zain N, Razali A. Phonophobia and hyperacusis: practical points from a case report. *Malays J Med Sci*. 2010;17(1):49–51.
11. Askenazy F, Dor E, Benoit M, Dupuis G, Serret S, Myquel M, Seddiki Y. Catatonia in a 14-year-old girl: treatment with clorazepam and carbamazepine, a 10-year follow-up. Encephale. 2010 Feb;36(1):46-53.
12. Attree EA, Dancey CP, Keeling D, Wilson C. Cognitive Function in People with Chronic Illness: Inflammatory Bowel Disease and Irritable Bowel Syndrome, Applied Neuropsychology. 2003. 10:2, 96-104, DOI: 10.1207/S15324826AN1002_05
13. Aucott, J.N., Rebman, A.W., Crowder, L.A. et al. Post-treatment Lyme disease syndrome symptomatology and the impact on life functioning: is there something here? Qual Life Res (2013) 22: 75. https://doi.org/10.1007/s11136-012-0126-6
14. Banerjee R, Liu JJ, Minhas HM. Lyme neuroborreliosis presenting with alexithymia and suicide attempts. J Clin Psychiatry. 2013 Oct;74(10):981.
15. Bar KJ, Jochum T, Hager F, Meissner W, Sauer H. Painful hallucinations and somatic delusions in a patient with the possible diagnosis of neuroborreliosis. Clin J Pain. 20 Jul-Aug;21(4):362-3.
16. Barr WB, Rastogi R, Ravdin L, Hilton E. Relations Among Indexes of Memory Disturbance and Depression in Patients With Lyme Borreliosis, Applied Neuropsychology, 1999 6:1, 12-18, DOI: 10.1207/s15324826an0601_2
17. Barnett W, Sigmund D, Roelcke U, Mundt C. Endogenous paranoid-hallucinatory syndrome caused by Borrelia encephalitis Nervenarzt 1991 Jul;62(7):445-7[German]
18. Battaglia H, Alvarez G, Mercau A, Fay M, Campodonico M. Psychiatric symptomatology associated with presumptive Lyme disease: Clinical evidence. J Spiro Tick-Borne Dis 2000; 7: 22-25.
19. Bechter K. Diagnosis of infectious or inflammatory psychosyndromes. Open Neurol J. 2012; 6:113-118.
20. Binalsheikh IM, Griesemer D, Wang S, Alvarez-Altalef R. Lyme neuroborreliosis presenting as Alice in Wonderland syndrome. Pediatr Neurol. 2012; 46:185-186.

21. Belman AL, Iyer M, Coyle PK, Dattwyler R. Neurologic manifestations in children with North American Lyme disease. Neurology. 1993 Dec;43(12):2609-14.
22. Benke T, Gasse T, Hittmair-Delazer M, Schmutzhard E. Lyme encephalopathy: long- term neuropsychological deficits years after acute neuroborreliosis. Acta Neurol Scand. 1995 May;91(5):353-7.
23. Berman DS, Wenglin BD. Complaints attributed to chronic Lyme disease: depression or fibromyalgia? Am J Med. 1995 Oct;99(4):440.
24. Bertholon P, Cazorla C, Carricajo A, Oletski A, Laurent B. Bilateral sensorineural hearing loss and cerebellar ataxia in the case of late stage Lyme disease. Braz J Otorhinolaryngol. 2012 Dec;78(6):124.
25. Bertrand E, Szpak GM, Pilkowski E, Habib N, Lipczynska-Lojkowska W, Rudnicka A, Tylewska-Wierzbanowska S, Kulczycki J. Central nervous system infection caused by *Borrelia burgdorferi*. Clinico-pathological correlation of three post-mortem cases. Folia Neuropathol 1999;37:43-51.
26. Biesiada G, Czapiel J, Sobczyk-Krupiarz I, Garlicki A, Mach T. Neuroborreliosis with extrapyramidal symptoms: a case report. Pol Arch Med Wewn. 2008 May;118(5):314-7.
27. Bitam I, Raoult D. Other Tick-Borne Diseases in Europe. Curr Probl Dermatol. 2009;37:130-154. Epub 2009 Apr 8
28. Blanc F; GEBLY. Neurologic and psychiatric manifestations of Lyme disease. Med Mal Infect. 2007 Jul-Aug;37(7- 8):435-45. Epub 2007 Mar 9. Review.
29. Bloom BJ, Wyckoff PM, Meissner HC, Steere AC. Neurocognitive abnormalities in children after classic manifestations of Lyme disease. Pediatr Infect Dis J 1998; 17(3):189-96.
30. Bolivar P, Griffin H. Bolivar P, Griffin H. California Residents with Chronic Lyme Disease. August 2018 DOI: 10.13140/RG.2.2.11651.55840
31. Blum K, Modestino EJ, Febo M, Steinberg B, McLaughlin T, Fried L, Baron D, Siwicki D, Badgaiyan RD. Lyme and Dopaminergic Function: Hypothesizing Reduced Reward Deficiency Symptomatology by Regulating Dopamine Transmission. J Syst Integr Neurosci. 2017 May;3(3).
32. Borgermans L, Goderis G, Vandevoorde J, Devroey D. Relevance of chronic Lyme disease to family medicine as a complex multidimensional chronic disease construct: A systematic review. Int J Family Med. 2014;2014:138016.
33. Bransfield RC. Aggressiveness, violence, homicidality, homicide, and Lyme disease. Neuropsychiatr Dis Treat. 2018; 14: 693–713. doi: 10.2147/NDT.S155143
34. Bransfield R, Brand S, Sherr V. Treatment of patients with persistent symptoms and a history of Lyme disease. N Engl J Med. 2001 Nov 8;345(19):1424-5.
35. Bransfield RC. Suicide and Lyme and associated diseases. Neuropsychiatr Dis Treat. 2017; 13: 1575–1587. doi: 10.2147/NDT.S136137
36. Bransfield RC. The psychoimmunology of Lyme/tick-borne diseases and its association with neuropsychiatric symptoms. Open Neurol J. 2012; 6:88-93.
37. Bransfield RC, Wulfman JS, Harvey WT, Usman AI. The association between tick-borne infections, Lyme borreliosis and autism spectrum disorders. Med Hypotheses. 2008;70(5):967-74.
38. Bransfield RC. Case report: Lyme disease and complex partial seizures. J Spiro Tick- borne Dis. 1999; 6:123-125 .
39. Bransfield RC. Diagnosis, treatment, and prevention of Lyme disease. JAMA. 1998 Sep 23-30;280(12):1049; author reply 1051. DOI: 10.1001/jama.280.12.1049
40. Bransfield RC. Lyme disease, comorbid tick-borne diseases, and neuropsychiatric disorders. Psychiatric Times. 2007 Dec; 24(14)59-61
41. Bransfield RC. Neuropsychiatric Lyme Borreliosis: An Overview with a Focus on a Specialty Psychiatrist's Clinical Practice. Healthcare (Basel) 2018 Sep; 6(3): 104. doi: 10.3390/healthcare6030104
42. Bransfield RC. Preventable cases of autism: relationship between chronic infectious disease and neurological outcome. Pediatric Health. (2009) April 3(2).
43. Bransfield RC. Relationship of Inflammation and Autoimmunity to Psychiatric Sequelae in Lyme Disease. Psychiatric Ann. 2012 42(9):337-41
44. Bransfield RC. The diagnosis of Lyme disease. Hosp Pract (Minneap). 1996 Aug 15;31(8):35, 40.
45. Bransfield RC, Kuhn M. Autism and Lyme disease. JAMA. 2013 Aug 28;310(8):856-7.
46. Bransfield RC. The psychoimmunology of Lyme/tick-borne diseases and its association with neuropsychiatric symptoms. Open Neurol J. 2012; 6: 88-93.
47. Bransfield RC. Intrusive symptoms and infectious encephalopathies. Neurol Psychiatr Brain Res. 2016; 22(1): 3-4.

48. Bransfield RC. Can infections and immune reactions to them cause violent behavior? Neurology, Psychiatry and Brain Research. 2012-03. DOI: 10.1016/j.npbr.2012.02.006
49. Bransfield RC, Relationship of Inflammation and Autoimmunity to Psychiatric Sequelae in Lyme Disease Psychiatric Annals. 2012-09-01. DOI: 10.3928/00485713-20120906-07
50. Bransfield, R.C. Lyme disease, comorbid tick-borne diseases, and neuropsychiatric disorders. *Psychiatr. Times* **2007** 24, 59-62.
51. Breitschwerdt EB, Maggi RG, Cadenas MB, Vissotto de Paiva Diniz PP. A groundhog, a novel Bartonella sequence, and my father's death. Emerg Infect Dis. 2009 Aug;15(12): 2080-6.
52. Breitschwerdt EB, Greenberg R, Maggi RG, Mozayeni BR, Lewis A, Bradley JM. Bartonella henselae Bloodstream Infection in a Boy with Pediatric Acute-Onset Neuropsychiatric Syndrome. J Cent Nerv Syst Dis. 2019; 11: 1179573519832014. doi: 10.1177/1179573519832014
53. Breitschwerdt EB. Bartonellosis, One Health and all creatures great and small. Vet Dermatol. 2017 Feb;28(1):96-e21.
54. Breitschwerdt EB. Maggi RG, Nicholson WL, Cherry NA, Woods CW. Bartonella sp. bacteremia in patients with neurological and neurocognitive dysfunction. J Clin Microbiol. 2008;46(9):2856–2861.
55. Brinck T, Hansen K, Olesen J. Headache resembling tension-type headache as the single manifestation of Lyme neuroborreliosis. Cephalalgia. 1993 Jun;13(3):207-9.
56. Brown JS Jr. Geographic correlation of schizophrenia to ticks and tick-borne encephalitis. Schizophr Bull 1994;20(4):755-75.
57. Bujak DI, Weinstein A, Dornbush RL. Clinical and neurocognitive features of the post Lyme syndrome. 1996, 23(8):1392-1397.
58. Burakgazi AZ. Lyme disease -induced polyradiculopathy mimicking amyotrophic lateral sclerosis. Int J Neurosci. 2014 Nov;124(11):859-62.
59. David R. Burdge, David P. O'Hanlon, Experience at a Referral Center for Patients with Suspected Lyme Disease in an Area of Nonendemicity: First 65 Patients, *Clinical Infectious Diseases*, Volume 16, Issue 4, April 1993, Pages 558–560, https://doi.org/10.1093/clind/16.4.558
60. Burns RB, Hartman EE. A 58-year-old man with a diagnosis of chronic Lyme disease, 1 year later. JAMA. 2003 Dec 24;290(24):3247.
61. Caliendo MV, Kushon DJ, Helz JW. Delirium and Lyme disease. Psychosomatics. 1995 Jan-Feb;36(1):69-74.
62. Cameron DC. Medical Hypotheses. Clinical trials validate the severity of persistent Lyme disease symptoms. 2009, 72(2):153-6. https://doi.org/10.1016/j.mehy.2008.09.030
63. Cameron D, Gaito A, Harris N, Bach G, Bellovin S, Bock K, Bock S, Burrascano J, Dickey C, Horowitz R, Phillips S, Meer-Scherrer L, Raxlen B, Sherr V, Smith H, Smith P, ILADS Working Group. Evidence-based guidelines for the management of Lyme disease. Expert Review of Anti-infective Therapy [01 Jan 2004, 2(1 Suppl):S1-13] 2004/01. DOI: 10.1586/14789072.2.1.S1
64. Cameron D. Severity of Lyme disease with persistent symptoms. Insights from a double- blind placebo-controlled clinical trial. Minerva Med. 2008 Oct;99(5):489-96.
65. Cameron DJ Consequences of treatment delay in Lyme disease. J Eval Clin Pract. 2007 Jun;13(3):470-2.
66. Cameron DJ. Proof That Chronic Lyme Disease Exists. Interdiscip Perspect Infect Dis. 2010; 2010: 876450. doi: 10.1155/2010/876450
67. Carter CJ. Schizophrenia: A Pathogenetic Autoimmune Disease Caused by Viruses and Pathogens and Dependent on GenesJ Pathog. 2011; 2011: 128318. doi: 10.4061/2011/128318
68. Cees C. van den Wijngaard, Agnetha Hofhuis, Margriet G. Harms, Juanita A. Haagsma, Albert Wong, G.A. de Wit, Arie H. Havelaar, Anna K. Lugnér, Anita W.M. Suijkerbuijk, Wilfrid van Pelt, The burden of Lyme borreliosis expressed in disability-adjusted life years, *European Journal of Public Health*, Volume 25, Issue 6, December 2015, Pages 1071–1078, https://doi.org/10.1093/eurpub/ckv091
69. Chabria SB, Lawrason J Altered mental status, an unusual manifestation of early disseminated Lyme disease: A case report. J Med Case Reports. 2007 Aug 9;1:62.
70. Chan L, Reilly KM, Snyder HS. An unusual presentation of cat scratch encephalitis. J Emerg Med. 1995 Nov- Dec;13(6):769-72.
71. Chandra AM, Keilp JG, Fallon BA Correlates of Perceived Health-Related Quality of Life in Post-treatment Lyme Encephalopathy. Psychosomatics. 2013 Jul 9.
72. Chandra A et al. Anti-neural antibody reactivity in patients with a history of Lyme borreliosis and persistent symptoms. Brain Behav Immun. Author manuscript; available in PMC 2011 Aug 1.Published in final edited form as: Brain Behav Immun. 2010 Aug; 24(6): 1018–1024. doi: 10.1016/j.bbi.2010.03.

73. Chavda V, Patel S. Lyme Neuroborreliosis - The Mystifying Pitfall: Neuropathology and Current Therapeutics. Recent Pat Antiinfect Drug Discov. 2019 Mar 26. doi: 10.2174/1574891X14666190327114641.
74. Cheherama M, Zagardo MT, Koski CL Subarachnoid hemorrhage in a patient with Lyme disease. Neurology. 1997;48:520-523
75. Cleveland WL, DeLaPaz RL, Fawwaz RA, Challop RS. High-Dose Glycine Treatment of Refractory Obsessive-Compulsive Disorder and Body Dysmorphic Disorder in a 5-Year Period. Neural Plast. 2009; 2009: 768398. doi: 10.1155/2009/768398
76. Cintron R, Pachner AR. Spirochetal diseases of the nervous system. Curr Opin Neurol. 1994 Jun;7(3):217-22. Review.
77. Citera M, Freeman PR, Horowitz RI. Empirical validation of the Horowitz Multiple Systemic Infectious Disease Syndrome Questionnaire for suspected Lyme disease. Int J Gen Med. 2017; 10: 249–273. doi: 10.2147/IJGM.S140224
78. Clarissou J, Song A, Bernede C, Guillemot D, Dinh A, Ader F, Perronne C, Salomon J. Efficacy of a long-term antibiotic treatment in patients with a chronic Tick Associated Poly-organic Syndrome (TAPOS). Med Mal Infect. 2009 Feb;39(2):108-15. Epub 2009 Jan 4.
79. Coetzer BR. Obsessive-Compulsive Disorder following Brain Injury: A Review. The International Journal of Psychiatry in Medicine. 2004, 34(2) https://doi.org/10.2190/XENN-NNWT-7N2K-R26A
80. Corral I, Sanchis G, Garcia-Ribas G, Quereda C, Escudero R, de Blas G. Demyelinating polyradiculitis in neuroborreliosis. Neurologia. 1995 Feb;10:110-113
81. Cowley G, Underwood A.A disease in disguise. Lyme can masquerade as migraine, or as madness. Newsweek. 2004 Aug 23;144(8):62.
82. Coyle PK, Deng Z, Schutzer SE, Belman AL, Benach J, Krupp LB, Luft B. Detection of Borrelia burgdorferi antigens in cerebrospinal fluid. Neurology 1993;43:1093-1097.
83. Coyle PK, Schutzer SE, Deng Z, Krupp LB, Belman AL, Benach JL, Luft BJ. Detection of Borrelia burgdorferi-specific antigen in antibody-negative cerebrospinal fluid in neurologic Lyme disease. Neurology. 1995 Nov;45(11):2010-5.
84. Créange A. Clinical manifestations and epidemiological aspects leading to a diagnosis of Lyme borreliosis: neurological and psychiatric manifestations in the course of Lyme borreliosis Med Mal Infect. 2007 Jul-Aug;37(7-8):532-9. Epub 2007 Mar 26.
85. Császár T, Patakfalvi A. Differential diagnostic problems in Lyme disease - Borrelia infection resulting in acute exogenous psychosis. Orv Hetil. 1994 Oct 9;135(41):2269-71.
86. Dattwyler RJ, Halperin JJ. Failure of tetracycline therapy in early Lyme disease. Arthritis Rheum. 1987 Apr;30(4):448- 50.
87. Dekonenko EP, Umanskii KG, Virich IE, Kupriianova LV, Rudometov, IuP, Bagrov FI. The basic syndromes of neurological disorders in Lyme borreliosis. Ter Arkh 1995; 67 (11) : 52-53
88. DeLuca J, Johnson SK, Beldowicz D, et al. Neuropsychological impairments in chronic fatigue syndrome, multiple sclerosis, and depression. *Journal of Neurology, Neurosurgery & Psychiatry* 1995;58:38-43.
89. Dersch R, Rauer S. Neuroborreliosis - Diagnostics, treatment and course. Nervenarzt. 2017 Apr;88(4):419-431. doi: 10.1007/s00115-016-0263-1.
90. Dersch R, Sarnes AA, Maul M. Quality of life, fatigue, depression and cognitive impairment in Lyme neuroborreliosis. Journal of Neurology, 2015, 262(11):2572
91. Donta ST, Noto RB, Vento JA. SPECT Brain Imaging in Chronic Lyme Disease. Clinical Nuclear Medicine & Volume 37, Number 9, September 2012
92. Druschky K, Stefan H, Grehl H, Neundörfer B. Secondary normal pressure hydrocephalus. A complication of chronic neuroborreliosis. Nervenarzt. 1999 Jun;70(6):556-9. German.
93. Dupeyron A, Lecocq J, Jaulhac B, et al. Sciatica, disk herniation, and neuroborreliosis. A report of four cases. Joint Bone Spine. 2004; 71: 433-437.
94. Dupuis MJ. Multiple neurologic manifestations of Borrelia burgdorferi infection. Rev Neurol (Paris) 1988;144(12):765- 75.
95. Edelstyn NM, Hunter B, Ellis SJ. Bilateral dorsolateral thalamic lesions disrupts conscious recollection. Neuropsychologia. 2006;44(6):931-8. Epub 2005 Oct 25.
96. Breitschwerdt EB, Mascarelli PE, Schweickert LA, Ricardo G. Maggi RG, Hegarty BC, Bradley JM, Woods CW. Hallucinations, sensory neuropathy, and peripheral visual deficits in a young woman infected with Bartonella koehlerae. J Clin Microbiol. 2011; 49: 3415–3417
97. Eikeland R, Mygland A, Herlofson K, Ljøstad U. European neuroborreliosis: quality of life 30 months after

treatment. Acta Neurol Scand. 2011Nov;124(5):349-54.
98. Elkins LE, Pollina DA, Scheffer SR, Krupp LB. Psychological states and neuropsychological performances in chronic Lyme disease. Appl Neuropsychol 1999;6(1):19-26.
99. Engman M-L, Lindström K, Sallamba M, Hertz C, et al. One-year follow-up of tick-borne central nervous system infections in childhood. Pediatric Infect Dis J. 2012; 31(6): 570-4.
100. Eskow E, Rao RV, Mordechai E. Concurrent infection of the central nervous system by Borrelia burgdorferi and Bartonella henselae: evidence for a novel tick-borne disease complex. Arch Neurol. 2001 Sep;58(9):1357-63.
101. Etienne M, Carvalho P, Fauchais AL, Pestel-Caron M, Doucet J, Chassagne P. Lyme neuroborreliosis revealed as a normal pressure hydrocephalus: a cause of reversible dementia. J Am Geriatr Soc. 2003 Apr;51(4):579-80.
102. Fallon BA, Keilp JG, Corbera KM, Petkova E, Britton CB, Dwyer E, Slavov I, Cheng J, Dobkin J, Nelson DR, Sackeim HA. A randomized, placebo-controlled trial of repeated IV antibiotic therapy for Lyme encephalopathy. Neurology Mar 2008, 70 (13) 992-1003; DOI: 10.1212/01.WNL.0000284604.61160.2d
103. Fallon BA, Schwartzberg M, Bransfield R, Zimmerman B, Scotti A, Weber CA, Liebowitz MR. Late-stage neuropsychiatric Lyme borreliosis. Case reports. Psychosomatics 1995; 36: 295-300.
104. Fallon BA, Das S, Plutchok JJ, Tager F, Liegner K, Van Heertum R. Functional brain imaging and neuropsychological testing in Lyme disease. Clin Infect Dis 1997; 25 (suppl 1): S57-S63.
105. Fallon BA, Sotsky J. Conquering Lyme Disease (Book) Columbia University Press, 2018
106. Fallon BA, Bird H, Hoven C, Cameron D, Liebowitz MR, Shaffer S. Psychiatric aspects of Lyme disease in children and adolescents: A community epidemiologic study in Westchester, New York. J Spiro Tick-Borne Dis 1994; 1:98-100
107. Fallon BA, Das S, Plutchok JJ, Tager F, Liegner K, Van Heertum R. Functional Brain Imaging and Neuropsychological Testing in Lyme Disease. Clin Infect Dis 1997; 25:S57- 63
108. Fallon BA, Javitch JA, Hollander E, Liebowitz MR. Hypochondriasis and obsessive compulsive disorder: overlaps in diagnosis and treatment. J Clin Psychiatry. 1991: 52(11):457-60.
109. Fallon BA, Keilp J, Prohovnik I, Heertum RV, Mann JJ. Regional cerebral blood flow and cognitive deficits in chronic Lyme disease. J Neuropsychiatry Clin Neurosci. 2003 Summer;15(3):326-32.
110. Fallon BA, Kochevar JM, Gaito A, Nields JA. The underdiagnosis of neuropsychiatric Lyme disease in children and adults. Psychiatr Clin North Am. 1998; 21: 693-703
111. Fallon BA, Levin ES, Schweitzer PJ, Hardesty D. Inflammation and central nervous system Lyme disease. Neurobiol Dis. 2010 Mar;37(3):534-41.
112. Fallon BA, Lipkin RB, Corbera KM, Yu S, Nobler MS, Keilp JG, Petkova E, Lisanby SH, Moeller JR, Slavov I, Van Heertum R, Mensh BD, Sackeim HA. Regional cerebral blood flow and metabolic rate in persistent Lyme encephalopathy. Arch Gen Psychiatry. 2009 May;66(5):554-63.
113. Fallon BA, Nields JA Acute disseminated encephalomyelitis [letter]. J Neuropsychiatry Clin Neurosci 1998 Summer;10(3):366-7
114. Fallon BA, Nields JA, Burrascano JJ, Liegner K, DelBene D, Liebowitz MR. The neuropsychiatric manifestations of Lyme Borreliosis. Psychiatric Quarterly 1992; 63: 95- 117.
115. Fallon BA, Nields JA, Parsons B, Liebowitz MR, Klein DF. Psychiatric manifestations of Lyme borreliosis. J Clin Psychiatry 1993 Jul;54(7):263-8
116. Fallon BA, Nields JA. Lyme Disease: A neuropsychiatric illness. Am J Psychiatry 1994 Nov;151(11):1571-83
117. Fallon BA, Petkova E, Keilp JG, Britton CB. Ongoing discussion about the US clinical Lyme trials. Am J Med. 2014 Feb;127(2):e7.
118. Fallon BA, Keilp JG, Corbera KM, Petkova K, Britton CB, Dwyer E, et al. A randomized, placebo-controlled trial of repeated IV antibiotic therapy for Lyme encephalopathy. Neurology 2008; 70: 992-1003.
119. Fallon BA, Petkova E, Keilp JG, Britton CB. A reappraisal of the U.S. clinical trials of post-treatment Lyme disease syndrome. Open Neurol J. 2012; 6:79-87.
120. Fallon BA, Schwartzberg M, Bransfield R, Zimmerman B, Scotti A, Weber CA, Liebowitz MR. Late-stage neuropsychiatric Lyme borreliosis: Differential diagnosis and treatment. Psychosomatics 1995;36:295-300
121. Fallon BA, Vaccaro B, Romano M, Clemente D. Neuropsychiatric and neuropathologic aspects of Lyme disease. Psychiatric Annals. 2006;36:120-128.
122. Fallon BA, Weis N, Tager F, Fein L, Liegner K, Liebowitz MR. Repeated antibiotic therapy in chronic Lyme disease. J Spiro Tick-Borne Dis. 1999; 6: 1-9.
123. Fallon BA, Keilp J, Prohovnik I, Heertum RV, Mann JJ. Regional cerebral blood flow and cognitive deficits

in chronic Lyme disease. J Neuropsychiatry Clin Neurosci 2003; 15: 326-332.
124. Farshad-Amacker NA, Scheffel H, Frauenfelder T, Alkadhi H. Brainstem abnormalities and vestibular nerve enhancement in acute neuroborreliosis. BMC Research Notes 2013; 6: 551.
125. Ferroir JP, Reignier A, Nicolle MH, Guillard A. Meningoradiculoencephalitis in Lyme disease. A case with major regressive mental disorders. Presse Med. 1988 Apr 16;17(14):697.
126. Flegr J1, Preiss M, Balátová P. Depressiveness and Neuroticism in Bartonella Seropositive and Seronegative Subjects-Preregistered Case-Controls Study. *Front Psychiatry.* 2018 Jul 13;9:314. doi: 10.3389/fpsyt.2018.00314. eCollection 2018.
127. Fritzsche M. Seasonal correlation of sporadic schizophrenia to Ixodes ticks and Lyme borreliosis. Int J Health Geogr. 2002; 1:2
128. Fritzsche M. Geographical and seasonal correlation of multiple sclerosis to sporadic schizophrenia. Int J Health Geogr. 2002 Dec 20;1(1):5.
129. Frykholm BO. On the question of infectious aetiologies for multiple sclerosis, schizophrenia and the chronic fatigue syndrome and their treatment with antibiotics. Med Hypotheses 2010 Apr;74(4):758-60.
130. Garakani A, Mitton AG. New-onset panic, depression with suicidal thoughts, and somatic symptoms in a patient with a history of Lyme disease. Case Rep Psychiatry. 2015;2015:457947.
131. Garcia-Monco JC, Benach JL. Lyme neuroborreliosis. Ann Neurol 1995 Jun; 37: 691-70.
132. García-Moncó JC, Benach JL Neurological manifestations of Lyme disease. Enferm Infec Microbiol Clin. 1989 Nov;7(9):501-6.
133. Garcia-Monco JC, Villar BF, Alen JC, Benach JL. Borrelia burgdorferi in the central nervous system: experimental and clinical evidence for early invasion. J Infect Dis. 1990 Jun;161(6):1187-93.
134. García-Moreno JM, Izquierdo G, Chacón J, Angulo S, Borobio MV. Neuroborreliosis in a patient with progressive supranuclear paralysis. An association or the cause? Rev Neurol. 1997 Dec;25(148):1919-21.
135. Gaudet EM, Gould ON, Lloyd V. Parenting When Children Have Lyme Disease: Fear, Frustration, Advocacy. Healthcare (Basel). 2019 Aug 8;7(3). pii: E95. doi: 10.3390/healthcare7030095.
136. Gasse T, Murr C, Meyersbach P, Schmutzhard E, Wachter H, Fuchs D. Neopterin production and tryptophan degradation in acute Lyme neuroborreliosis versus late Lyme encephalopathy. Eur J Clin Chem Clin Biochem. 1994 Sep;32(9):685-9.
137. Gaudino EA, Coyle PK, Krupp LB. Post-Lyme syndrome and chronic fatigue syndrome. Neuropsychiatric similarities and differences. Arch Neurol. 1997 Nov;54(11):1372-6.
138. Gentile I, Zappulo E, Militerni R, Pascotto A, Borgia G, Bravaccio C. Etiopathogenesis of autism spectrum disorders: Fitting the pieces of the puzzle together. Med Hypotheses. 2013 Jul;81(1):26-35.
139. Gerber A, Zalneraitis L. Childhood neurologic disorders and Lyme disease during pregnancy. Pediatric Neurology. 1994 11(1):41-44 https://doi.org/10.1016/0887-8994(94)90088-4
140. Geschwind MD, Josephs KA, Parisi JE, B. Keegan M. A 54-year-old man with slowness of movement and confusion. Neurology. Author manuscript; available in PMC 2009 Feb 6.Published in final edited form as: Neurology. 2007 Nov 6; 69(19): 1881–1887. doi: 10.1212/01.wnl.0000290370.14036.69
141. Geschwind MD, Shu H, Haman A, Sejvar JJ, Miller BL. Rapidly Progressive Dementia. Ann Neurol. Author manuscript; available in PMC 2009 Feb 25. Published in final edited form as: Ann Neurol. 2008 Jul; 64(1): 97–108. doi: 10.1002/ana.21430
142. Geschwind MD, Haman A, Miller BL. Rapidly Progressive Dementia. Neurol Clin. Author manuscript; available in PMC 2009 Jul 6.Published in final edited form as: Neurol Clin. 2007 Aug; 25(3): 783–vii. doi: 10.1016/j.ncl.2007.04.001
143. George TI, Manley G, Koehler JE, Hung VS, McDermott M, Bollen A. Detection of Bartonella henselae by polymerase chain reaction in brain tissue of an immunocompromised patient with multiple enhancing lesions. Case report and review of the literature. J Neurosurg. 1998 Oct;89(4):640-4.
144. Gerstenblith TA, Stern TA. Lyme disease: a review of its epidemiology, evaluation, and treatment. Psychosomatics. 2014 Sep-Oct;55(5):421-9.
145. Gheorghiev C, De Montleau F, Defuentes G. Alcohol and epilepsy: A case report between alcohol withdrawal seizures and neuroborreliosis. Encephale 2011 Jun;37(3):231-7.
146. GIANNUZZI TR, S. DAINI S, L. BERNARDINI L, L. PETRONGOLO L, SILVER NG. Psychiatric emergencies (part III): psychiatric symptoms resulting from organic diseases. Eur Rev Med Pharmacol Sci. 013;17(Suppl 1):86-99.
147. Greenberg HE, Ney G, Scharf SM, Ravdin L, Hilton E. Sleep quality in Lyme disease. Sleep. 1995 Dec;18(10):912-6.

148. Greenberg R. Commentary Re: a critical appraisal of the mild axonal peripheral neuropathy of late neurologic Lyme disease, by Wormser et al. DMID 2016. Diagn Microbiol Infect Dis. 2017 May;88(1):106. doi: 10.1016/j.diagmicrobio.2017.01.017. Epub 2017 Feb 8. PubMed PMID: 28365059.
149. Greenberg R. Infections and childhood psychiatric disorders: Tick-borne illness and bipolar disorder in youth. Bipolar Disord. 2017; 3:1.
150. Greenberg R. The Role of Infection and Immune Responsiveness in a Case of Treatment-Resistant Pediatric Bipolar Disorder. Front. Psychiatry, 22 May 2017 | https://doi.org/10.3389/fpsyt.2017.00078
151. Greenblatt D, Krupp LB, Belman AL Parainfectious meningo-encephalo- radiculo-myelitis (cat scratch disease, Lyme borreliosis, brucellosis, botulism, legionellosis, pertussis, mycoplasma). Handb Clin Neurol 2013; 112: 1195-207.
152. Grzywa A, Karakuła H, Górecka J, Chuchra M. Delusional disorders in the course of tick-born encephalitis and borreliosis in patients with hemophilia A and posttraumatic epilepsy--diagnostic and therapeutic difficulties Pol Merkur Lekarski. 2004 Jan;16(91):60- 3. Polish.
153. Gueglio B, Raffi F, Marjolet M. Lyme neuroborreliosis of mental manifestation. Apropos of a case. Rev Med Interne. 1996;17(7):599. [French]
154. Gustaw K, Beltowska K, Dlugosz E. Co-existance of toxoplasmosis and neuroborreliosis - a case report. Ann Agric Environ Med. 2005;12(2):305-8.
155. Gustaw K, Beltowska K, Studzinska MM. Neurological and psychological symptoms after the severe acute neuroborreliosis. Ann Agric Environ Med 2001;8(1):91-4
156. Gustaw-Rothenberg K. Cognitive impairments after tick-borne encephalitis. Dement Geriatr Cogn Disord. 2008;26:165-168.
157. Haass A. Lyme neuroborreliosis. Curr Opin Neurol. 1998;11:253-258.
158. Hájek T, Libiger J, Janovská D, Hájek P, Alda M, Höschl C. Clinical and demographic characteristics of psychiatric patients seropositive for Borrelia burgdorferi. Eur Psychiatry. 2006 Mar;21(2):118-22.
159. Hajek T, Paskova B, Janovska D, Bahbouh R, Hajek P, Libiger J, Hoschl C. Higher prevalence of antibodies to Borrelia burgdorferi in psychiatric patients than in healthy subjects. Am J Psychiatry 159:297-301, February 2002.
160. Halperin JJ, Luft BJ, Anand AK, Roque CT, Alvarez O, Volkman DJ, Dattwyler RJ. Lyme neuroborreliosis: central nervous system manifestations. Neurology. 1989 Jun;39(6):753-9.
161. Halperin JJ. Prolonged Lyme disease treatment: enough is enough. Neurology 2008; 70(13): 986-987.
162. Harvey WT, Martz D. Motor neuron disease recovery associated with IV ceftriaxone and anti-Babesia therapy. Acta Neurol Scand 2007: 115: 129–131.
163. Helon B, Tluczek TW, Buczyjan A, Adamczyk-Helon A, Wojnarowicz M, Mikula R, Cicinski P, Bojarska J. Polymorphic mental disorders in the course of Lyme borreliosis--case study. Psychiatr Pol. 2009 May-Jun;43(3):353-61.
164. Hernandez-Albujar S, Rubio G, Gopar J, Galeote G, Rey R, Gil A. Parasitic delirium in patient with multiorganic pathology: a complex situation. An Med Interna 1996 Nov;13(11):549-51 [Spanish]
165. Hess A, Buchmann J, Zettl UK, Henschel S, Schlaefke D, Grau G, Benecke R. Borrelia burgdorferi central nervous system infection presenting as an organic schizophrenialike disorder. Biol Psychiatry 1999 Mar 15;45(6):795
166. Hildenbrand P, Craven DE, Jone R et al. Lyme Neuroborreliosis: Manifestations of a rapidly emerging zoonosis. Am J Neuroradiol 2009;30:1079–87.
167. Hinze-Selch D. Infection, treatment and immune response in patients with bipolar disorder versus patients with major depression, schizophrenia or healthy controls. Bipolar Disorders. First published: 04 September 2002, 4(1):81-3. https://doi.org/10.1034/j.1399-5618.4.s1.32.x
168. Hodgson R, Belgamwar R, Al-tawarah Y, MacKenzie G The use of atypical antipsychotics in the treatment of schizophrenia in North Staffordshire. Hum Psychopharmacol. 2005 Mar;20(2):141-7.
169. Holtze M, Mickiene´A. Atlas A, Lindquist L, Schwieler L. Elevated cerebrospinal fluid kynurenic acid levels in patients with tick-borne encephalitis. J Intern Med. 2012; 272: 394–401.
170. Horowitz RI, Freeman PR. Precision medicine: retrospective chart review and data analysis of 200 patients on dapsone combination therapy for chronic Lyme disease/post-treatment Lyme disease syndrome: part . Int J Gen Med. 2019; 12: 101–119. doi: 10.2147/IJGM.S193608
171. Horowitz RI, Freeman PR. Precision Medicine: The Role of the MSIDS Model in Defining, Diagnosing, and Treating Chronic Lyme Disease/Post Treatment Lyme Disease Syndrome and Other Chronic Illness: Part 2. Healthcare (Basel) 2018 Dec; 6(4): 129. Published online 2018 Nov 5. doi: 10.3390/healthcare6040129

172. Horst-G. Markus M, Rojewski MT, Schmitt A, Tumani H, Bechter K, Schmitt M. Flow cytometric analysis of T cell subsets in paired samples of cerebrospinal fluid and peripheral blood from patients with neurological and psychiatric disorders. Brain, Behavior, and Immunity, Volume 23, Issue 1, 2009, Pages 134-142, ISSN 0889-1591, https://doi.org/10.1016/j.bbi.2008.08.003.
173. Hovius JWR et al. A case of meningoencephalitis by the relapsing fever spirochaete Borrelia miyamotoi in Europe. Lancet 2013 Aug 17;382:658.
174. Hurley RA, Taber KH. Acute and chronic Lyme disease: Controversies for neuropsychiatry. J Neuropsychiatry Clin Neurosci 2008;20(1):iv-6.
175. Iero I, Elia M, Cosentino FI, Lanuzza B, Spada RS, Toscano G, Tripodi M, Belfiore A, Ferri R. Isolated monolateral neurosensory hearing loss as a rare sign of neuroborreliosis. Neurol Sci. 2004 Apr;25(1):30-3.
176. Imai DM, Barr BC, Daft B, Bertone JJ, Feng S, Hodzic E, Johnston JM, Olsen KJ, Barthold SW. Lyme neuroborreliosis in two horses. Vet Pathol 2011; 48: 1151-1157.
177. Issakainen J, Gnehm HE, Lucchini GM, Zbinden R Value of clinical symptoms, intrathecal specific antibody production and PCR in CSF in the diagnosis of childhood Lyme neuroborreliosis. Klin Padiatr 1996 May-Jun; 208: 106-109.
178. Izquierdo G, Aguilar J, Barranquero A, Navarro G, Borobio MV, Angulo S, Domínguez I, Quesada MA. Positive anti-Borrelia antibodies in patients with clinical manifestations compatible with neuroborreliosis. Neurologia. 1992 Feb;7(2):50-4. Spanish.
179. Jacek E, Fallon BA, Chandra A, Crow MK, Wormser GP, Alaedini A. Increased IFNα activity and differential antibody response in patients with a history of Lyme disease and persistent cognitive deficits. J Neuroimmunol. 2013 Feb 15;255(1-2):85- 91.
180. James FM, Engiles JB, Beech J. Meningitis, cranial neuritis, and radiculoneuritis associated with Borrelia burgdorferi infection in a horse. J Am Vet Med Assoc 2010; 237: 1180-1185.
181. Jarskog LF, Mattioli MA, Perkins DO, Lieberman JA. First-episode psychosis in a managed care setting: clinical management and research. Am J Psychiatry. 2000 Jun;157(6):878-84.
182. Johnco C, Kugler BB, Murphy TK, Storch EA. Obsessive-compulsive symptoms in adults with Lyme disease. Gen Hosp Psychiatry. 2018 Jan 31;51:85-89. doi: 10.1016/j.genhosppsych.2018.01.009.
183. Johnson L, Wilcox S, Mankoff J, Stricker RB. Severity of chronic Lyme disease compared to other chronic conditions: a quality of life survey. PeerJ. 2014 Mar 27;2:e322. doi: 10.7717/peerj.322.
184. Jovanovic J, Cvjetkovic D, Vukadinov J. Lyme disease--neuroborreliosis. Med Pregl. 1995;48(3-4):120-2.
185. Juchnowicz D, Rudnik I, Czernikiewicz A, Zajkowska J, Pancewicz SA. Mental disorders in the course of Lyme borreliosis and tick-borne encephalitis. Przegl Epidemiol 2002;56 Suppl 1:37-50 [Polish]
186. Judy L. Thompson, Nina Urban, Mark Slifstein, Xiaoyan Xu, Lawrence S. Kegeles, Ragy R. Girgis, Yael Beckerman, Jill M. Harkavy-Friedman, Roberto Gil, Anissa Abi-Dargham. Striatal Dopamine Release in Schizophrenia Comorbid with Substance DependenceMol Psychiatry. 2013 Aug; 18(8): 909–915. doi: 10.1038/mp.2012.109
187. Kaiser B. Neuroborreliosis. J Neurol. 1998; 245:247-255
188. Kanjwal K, Karabin B, Kanjwal Y, Grubb BP. Postural orthostatic tachycardia syndrome following Lyme disease. Cardiol J. 2011;18(1):63-6.
189. Kaplan A. Neuropsychiatric masquerades. Psychiatr Times 2009 Feb; 26(2):1-8.
190. Kaplan RF, Jones-Woodward L, Workman K, Steere AC, Logigian EL, Meadows ME. Neuropsychological deficits in Lyme disease patients with and without other evidence of central nervous system pathology. Appl Neuropsychol. 1999;6(1):3-11.
191. Kaplan RF, Jones-Woodward L. Lyme encephalopathy: a neuropsychological perspective. Semin Neurol. 1997 Mar;17(1):31-7.
192. Kaplan RF, Meadows ME, Vincent LC, Logigian EL, Steere AC. Memory impairment and depression in patients with Lyme encephalopathy: comparison with fibromyalgia and nonpsychotically depressed patients. Neurology. 1992 Jul;42(7):1263-7.
193. Karma A, Stenborg T, Summanen P, Immonen I, Mikkila H, and Seppala I. Long-term follow-up of chronic Lyme neuroretinitis. Retina 1996; 16: 505-509.
194. Karma A, Pirttilä TA, Viljanen MK, Lähde YE, Raitta CM. Secondary retinitis pigmentosa and cerebral demyelination in Lyme borreliosis. Br J Ophthalmol. 1993 Feb;77(2):120-2.
195. Karma A, Seppälä I, Mikkilä H, Kaakkola S, Viljanen M, Tarkkanen A. Diagnosis and clinical characteristics of ocular Lyme borreliosis. Am J Ophthalmol. 1995 Feb;119(2):127-35.
196. Karrasch M, Fingerle V, Boden K, Darr A, Baier M, Straube E, Nenadic I. Neuroborreliosis and acute

encephalopathy: The use of CXCL13 as a biomarker in CNS manifestations of Lyme borreliosis. Ticks Tick Borne Dis. 2018 Feb;9(2):415-417. doi: 10.1016/j.ttbdis.2017.
197. Keilp JG, Corbera K, Gorlyn M, Oquendo MA, Mann JJ, Fallon BA. Neurocognition in Post-Treatment Lyme Disease and Major Depressive Disorder. Arch Clin Neuropsychol. 2018 Nov 12.
198. Keilp JG, Corbera K, Slavov I, Taylor MJ, Sackeim HA, Fallon BA. WAIS-III and WMS-III performance in chronic Lyme disease. J Int Neuropsychol Soc. 2006 Jan;12(1):119-29.
199. Keller TL, Halperin JJ, and Whitman M. PCR detection of Borrelia burgdorferi DNA in cerebrospinal fluid of Lyme neuroborreliosis patients. Neurology 1992; 43: 32- 42.
200. Kennedy PGE, VIRAL ENCEPHALITIS: CAUSES, DIFFERENTIAL DIAGNOSIS, AND MANAGEMENT *Journal of Neurology, Neurosurgery & Psychiatry* 2004;75:i10-i15.
201. Kepa L, Oczko-Grzesik B, Badura-Glombik T. Evaluation of cerebrospinal fluid serotonin (5-HT) concentration in patients with post-Lyme disease syndrome-- preliminary study Przegl Epidemiol. 2008;62(4):793-800. Polish.
202. Kobayashi K, Mizukoshi C, Aoki T, Muramori F, Hayashi M, Miyazu K, Koshino Y, Ohta M, Nakanishi I, Yamaguchi N. Borrelia burgdorferi-seropositive chronic encephalomyelopathy: Lyme neuroborreliosis? An autopsied report. Dement Geriatr Cogn Disord. 1997 Nov-Dec;8(6):384-90.
203. Kohler J, Kern U, Kasper J, Rhese-Küpper B, Thoden U. Chronic central nervous system involvement in Lyme borreliosis. Neurology. 1988 Jun;38(6):863-7.
204. Kohler J. Lyme borreliosis in neurology and psychiatry. Fortschr Med. 1990 Apr 10;108(10):191-3, 197. Review. [German]
205. Kollikowski HH, Schwendemann G, Schulz M, Wilhelm H, Lehmann HJ. Chronic borrelia encephalomyeloradiculitis with severe mental disturbance: immunosuppressive versus antibiotic therapy. J Neurol. 1988 Jan;235(3):140-2.
206. Koola MM et al. Undiagnosed Lyme disease in adults with schizophrenia. Schizophrenia Research. 2015 Oct;168(1-2):579-80.
207. Krause DL, Norbert Müller N. The relationship between Tourette's syndrome and infections. Open Neurol J. 2012; 6: 124-128.
208. Kristensson K. Microbes' roadmap to neurons. Nat Rev Neurosci. 2011 Jun;12(6):345-57.
209. Kristoferitsch W. Neurologice manifestations in Lyme borreliosis. 1993, 11(3):393–400.
210. Krüger H, Heim E, Schuknecht B, Scholz S. Acute and chronic neuroborreliosis with and without CNS involvement: a clinical, MRI, and HLA study of 27 cases. JNeurol. 1991 Aug;238(5):271-80.
211. Krupp LB, Hyman LG, Grimson R, Coyle PK, Melville P, Ahnn S, Dattwyler R, Chandler B. Study and treatment of post Lyme disease (STOP-LD): a randomized double masked clinical trial. Neurology. 2003 Jun 24;60(12):1923-30.
212. Krupp LB, Masur D, Schwartz J, Coyle PK, Langenbach LJ, Fernquist SK, Jandorf L, Halperin JJ. Cognitive functioning in late Lyme borreliosis. Arch Neurol. 1991 Nov;48(11):1125-9.
213. Krupp LB, Masur D, Schwartz J, Coyle PK, Langenback IJ, Fernquist SK. Cognitive functioning in late Lyme borreliosis. Arch Neurol 1999; 48: 1125-1129.
214. Kuhn M, Bransfield RC. Divergent opinions of proper Lyme disease diagnosis and implications for children co-morbid with autism spectrum disorder. Med Hypotheses. 2014 Sep;83(3):321-5.
215. Kuhn M, Grave S, Bransfield R, Harris S. Long term antibiotic therapy may be an effective treatment for children co-morbid with Lyme disease and autism spectrum disorder. Med Hypotheses. 2012 May;78(5):606-15.
216. Lapenta J. Lapenta JM. Lyme Disease and Dementia, Alzheimer, Parkinson, Autism, an Easy Way to Destroy your Brain. Investigative Dermatology and Venereology Research. 2018;(1):30-43.https://www.ommegaonline.org/article-details/Lyme-disease-and-dementia,-Alzheimer,-Parkinson,-Autism,-an-easy-way-to-destroy-your-brain./1992?fbclid=IwAR0Y3i-H7k_-Rc4CYHZH8vTHiPvRm3H_50JACRWGoqNI_cGhQ3greuEv1Og#.XFT1ZLNXlmM.linkedin
217. Latov N, Wu AT, Chin RL, Sander HW, Alaedini A, Brannagran TH. Neuropathy and cognitive impairment following vaccination with the OspA protein of Borrelia burgdorferi. J Peripher Nerv Syst 2004; 9: 165-167.
218. Lawrence C, Lipton RB, Lowy RD, Coyle PK. Seronegative chronic relapsing neuroborreliosis. Eur Neurol 1995; 35(2): 113-117.
219. Leedy MJ, Jackson M, Callahan JL. Treating depression and compensatory narcissistic personality style in a man with chronic Lyme disease. Clinical Case Studies. 2007 Oct; 6(5):430-42.
220. Legatowicz-Koprowska M, Gziut AI, Walczak E, Gil RJ, Wagner T. Borreliosis-

-simultaneous Lyme carditis and psychiatric disorders--case report. Pol Merkur Lekarski. 2008 May;24(143):433-5. Polish.

205. Leonard L, Swedo SE, Garvey M et al. Postinfectious and Other Forms Obsessive Compulsive Disorder. Child and Adolescent Psychiatric Clinics of North America. 1999 8(3):497-511https://doi.org/10.1016/S1056-4993(18)30166-4

221. Leslie TA, Levell NJ, Cutler SJ, Cann KJ, Smith ME, Wright DJ, Gilkes JJ, Robinson TW. Acrodermatitis chronica atrophicans: a case report and review of the literature. Br J Dermatol. 1994 Nov;131(5):687-93.

222. Levenson JL Psychiatric issues in infectious diseases. Primary Psychiatry 2006;13(5):29-32.

223. Liegner KB, Duray P, Agricola M, Rosenkilde C, Yannuzzi LA, Ziska M, Tilton RC, Hulinska D, Hubbard J, Fallon BA. Lyme disease and the clinical spectrum of antibiotic responsive chronic meningoencephalomyelitides. J Spiro Tick-Borne Dis 1997; 4: 61-73.

224. Lino AMM, Spera RR, de Campos FPF, et al. Adult-onset opsoclonus-myoclonus-ataxia syndrome as a manifestation of Brazilian lyme disease-like syndrome: a case report and review of literature. *Autops Case Rep*. 2014;4(1):29–37. Published 2014 Mar 31. doi:10.4322/acr.2014.005

225. Listernick R. A 17-year-old boy previously diagnosed with chronic Lyme disease. Patient complained of low-grade fevers, headaches, pharyngitis, and suspected his mother was trying to poison him. Pediatr Ann. 2004 Aug;33(8):494-8.

226. Livengood JA and Gilmore RD, Jr. Invasion of human neuronal and glial cells by an infectious strain of Borrelia burgdorferi. Microbes and Infection. 2006; 8: 2832-2840.

227. Lobraico J, Butler A, Petrini J, Ahmadi R. New insights into stages of lyme disease symptoms from a novel hospital-based registry. J Prim Care Community Health. 2014 Oct;5(4):284-7.

228. Logigian EL, Johnson KA, Kijewski MF, Kaplan RF, Becker JA, Jones KJ, Garada BM, Holman BL, Steere AC. Reversible cerebral hypoperfusion in Lyme encephalopathy. Neurology 1997 Dec;49(6):1661-70.

229. Logigian EL, Kaplan RF, Steere AC Chronic neurologic manifestations of Lyme disease. N Engl J Med. 1990 Nov 22;323(21):1438-44.

230. Logigian EL, Kaplan RF, Steere AC. Successful treatment of Lyme encephalopathy with intravenous ceftriaxone. J Infect Dis. 1999 Aug;180(2):377-83.

231. Luft BJ, Steinman CR, Neimark HC, Muralidhar B, Rush T, Finkel MF, Kundel M, Dattwyler RJ. Invasion of the CNS by Borrelia burgdorferi in acute disseminated infection. JAMA 1992; 267: 1364-1367.

232. Maes M, Berk M, Goehler L, Song C et al. Depression and sickness behavior are Janus-faced responses to shared inflammatory pathways. BMC Med. 2012; 10: 66. doi: 10.1186/1741-7015-10-66

233. Maillefert JF, Dardel P, Piroth C, Tavernier C. Mental nerve neuropathy in Lyme disease. Rev Rhum Engl Ed. 1997 Dec;64(12):855.

234. Maimone D, Villanova M, Stanta G, Bonin S, Malandrini A, Guazzi GC, Annunziata P. Detection of Borrelia burgdorferi DNA and complement membrane attack complex deposits in the sural nerve of a patient with chronic polyneuropathy and tertiary Lyme disease. Muscle Nerve. 1997 Aug;20(8):969-75.

235. Markeljević J, Sarac H, Rados M. Tremor, seizures and psychosis as presenting symptoms in a patient with chronic Lyme neuroborreliosis (LNB). Coll Antropol. 2011 Jan;35 Suppl 1:313-8.

236. Mascarelli PE, Maggi RG, Hopkins S, Mozayeni BR, Trull CL, Bradley JM, Hegarty BC, Breitschwerdt EB Bartonella henselae infection in a family experiencing neurological and neurocognitive abnormalities after woodlouse hunter spider bites Parasites Vectors 2013; 6:98.

237. Matera G, Labate A, Quirino A, Lamberti AG, BorzÃ G, Barreca GS, Mumoli L, Peronace C, Giancotti A, Gambardella A, Foca A, Quattrone A. Chronic neuroborreliosis by B. garinii: an unusual case presenting with epilepsy and multifocal brain MRI lesions. New Microbiol. 2014 Jul;37(3):393-7

238. Matson JL, Rachel L. Goldin. Comorbidity and autism: Trends, topics and future directions. Research in Autism Spectrum Disorders, Volume 7, Issue 10, 2013, Pages 1228-1233, ISSN 1750-9467, https://doi.org/10.1016/j.rasd.2013.07.003.

239. Mattingley DW, Koola MM. Association of Lyme Disease and Schizoaffective Disorder, Bipolar Type: Is it Inflammation Mediated? *Indian J Psychol Med*. 2015;37(2):243–246. doi:10.4103/0253-7176.

240. Mattsson N, Bremell D, Anckarsater R, Blennow K, Anckarsater H, Zetterberg H, Hagberg L. Neuroinflammation in Lyme neuroborreliosis affects amyloid metabolism. BMC Neurol. 2010 Jun 22;10(1):51

241. McAuliffe P, Brassard MR, Fallon B. Memory and executive functions in adolescents with posttreatment Lyme disease. Applied Neuropsych. 2008; 15:208-219

242. Middelveen MJ, Stricker RS. Filament formation associated with spirochetal infection: a comparative

approach to Morgellons disease. Clin Cosmet Investig Dermatol. 2011; 4: 167–177. Published online 2011 Nov 14. doi: 10.2147/CCID.S26183

243. Middelveen MJ, Fesler MC, Stricker RB. History of Morgellons disease: from delusion to definition. Clin Cosmet Investig Dermatol. 2018; 11: 71–90. doi: 10.2147/CCID.S152343
244. Merlo A, Weder B, Ketz E, Matter L. Locked-in state in Borrelia burgdorferi meningitis. J Neurol. 1989;236:305-306.
245. Mervine P. Chronic lyme disease: psychogenic fantasy or somatic infection? *Environ Health Perspect*. 2003;111(2):A76–A77. doi:10.1289/ehp.111-a76
246. Mikkilä H, Seppälä I, Leirisalo-Repo M, Immonen I, Karma A. The etiology of uveitis: the role of infections with special reference to Lyme borreliosis. Acta Ophthalmol Scand. 1997 Dec;75(6):716-9.
247. Mikkilä HO, Seppälä IJ, Viljanen MK, Peltomaa MP, Karma A. The expanding clinical spectrum of ocular Lyme borreliosis. Ophthalmology. 2000 Mar;107(3):581-7.
248. Miklossy J, Donta S, Mueller K, Nolte O, Perry G. Chronic or late Lyme neuroborreliosis: Present and future. Open Neurology J. 2012; 6:78.
249. Millner M. Neurologic manifestations of Lyme borreliosis in children. Wien Med Wochenschr 1995; 145 (7-8): 178-182.
250. Möhrenschlager M, Köhn FM, Bauer M, Schaaf L, Hofmann H, Ring J. Late Lyme disease masking a non-functioning adenoma of the anterior lobe of the pituitary gland. Andrologia. 2002 Jun;34(3):162-3.
251. Mokry M, Flaschka G, Kleinert G, Kleinert R, Fazekas F, Kopp W. Chronic Lyme disease with an expansive granulomatous lesion in the cerebellopontine angle. Neurosurgery. 1990 Sep;27(3):446-51.
252. Montplaisir J, de Champlain J, Young SN, Missala K, Sourkes TL, Walsh J, Rémillard G. Narcolepsy and idiopathic hypersomnia. Neurology Nov 1982, 32 (11) 1299; DOI: 10.1212/WNL.32.11.1299
253. Morgen K, Martin R, Stone RD, Grafman J, Kadom N, McFarland HF, Marques FLAIR and magnetization transfer imaging of patients with post-treatment Lyme disease syndrome. Neurology. 2001 Dec 11;57(11):1980-5.
254. Morgenstern RG. Chronic Lyme disease: it's not all in our heads. *Environ Health Perspect*. 2003;111(2):A77. doi:10.1289/ehp.111-a77a
255. Morris G, Berk M, Walder K, Maes M. The putative role of viruses, bacteria, and chronic fungal biotoxin exposure in the genesis of intractable fatigue accompanied by cognitive and physical disability. Mol Neurobiol. 2016 May;53(4):2550-71.
256. Moses JM, RS Riseberg, and JM Mansbach. Lyme disease presenting with persistent headache. Pediatrics 2003; 112: 477-449.
257. Muller M, Retzl J, Plank E, Scholz H, Ziervogel H, Stanek G. Prevalence of Borrelia burgdorferi serum antibodies in 651 patients with predominantly neurologic diseases. Wien Klin Wochenschr. 1993;105(21):599-602.
258. Muller N, Riedel M, Straube A, Gunther W, Wilske B. Increased anti- streptococcal antibodies in patients with Tourette's syndrome. Psychiatry Res. 2000 Apr 24;94(1):43-9.
259. Murray R, Morawetz R, Kepes J, el Gammal T, LeDoux M. Lyme neuroborreliosis manifesting as an intracranial mass lesion. Neurosurgery. 1992 May;30(5):769-73.
260. Nachman SA, Pontrelli L. Central nervous system Lyme disease. Seminars in Pediatric Infectious Diseases. 2003, 14(2):123-30. https://doi.org/10.1053/spid.2003.127229.
261. Newberg A, Hassan A, Alavi A. Cerebral metabolic changes associated with Lyme disease Nucl Med Commun 2002 August;23(8):773-777
262. Nicolson GL Chronic bacterial and viral Infections in neurodegenerative and neurobehavioral diseases. Lab Medicine. 2008;39(5):291-9.
263. Nicolson GL, Haier J. Role of chronic bacterial and viral infections in neurodegenerative, neurobehavioral, psychiatric, autoimmune and fatiguing illnesses. Br J Med Pract. 2009:2(4)20-8.
264. Nields JA, Fallon BA, Jastreboff PJ. Carbamazepine in the treatment of Lyme disease-induced hyperacusis. J Neuropsychiatry Clin Neurosci 1999 Winter;11(1):97-9
265. Nields JA, Fallon BA. Differential diagnosis and treatment of Lyme disease with special reference to psychiatric practice. Directions Psychiatry, 1998; 18: 209-228.
266. Nields JA, Kueton JF. Tullio phenomenon and seronegative Lyme borreliosis. Lancet. 1991 Jul 13;338:128-9
267. Nocton JJ, Bloom BJ, Rutledge BJ, Persing DJ, Logigian EL, Schmid CH, Steere AC. Detection of Borrelia burgdorferi DNA by polymerase chain reaction in cerebrospinal fluid in Lyme neuroborreliosis. J. Infect Dis 1996; 174: 623-627.

268. Oczko-Grzesik B, Kępa L, Puszcz-Matlińska M, Pudło R, Żurek A, Badura-Głąbik T. Estimation of cognitive and affective disorders occurrence in patients with Lyme borreliosis. Ann Agric Environ Med. 2017 Mar 1;24(1):33-38.
269. Oglodek E, Mos D, Araszkiewicz A. Coexisting of borreliosis, depression and psoriasis--case report. Pol Merkur Lekarski. 2010 Jan;28(163):53-5.
270. Oksi J, Kalimo H, Marttila RJ, Marjarnaki M, Sonninen P, Nikoskelainen J, Viljanen MK. Inflammatory brain changes in Lyme Borreliosis. A report on three patients and review of literature. Brain. 1996 Dec; 119 (Pt 6) : 2143-2154
271. Omasits M, Seiser A, Brainin M. Recurrent and relapsing course of borreliosis of the nervous system. Wien Klin Wochenschr. 1990 Jan 5;102(1):4-12. Review.
272. Pachner AR. Early disseminated Lyme disease: Lyme meningitis. Am J Med. 1995; 98(4A):30S-37S
273. Pachner AR. Borrelia burgdorferi in the nervous system: the new "great imitator". Ann N Y Acad Sci. 1988;539:56-64.
274. Pachner AR, Duray P, Steere AC. Central nervous system manifestations of Lyme disease. Arch Neurol. 1989 Jul;46(7):790-5.
275. Pachner AR, Steiner I. Lyme neuroborreliosis: infection, immunity and inflammation. Lancet Neurol 2007; 6:544- 52.
276. Pachner AR. Neurologic manifestations of Lyme disease, the new "great imitator". Rev Infect Dis. 1989 Sep-Oct;11 (Suppl 6):S1482-6.
277. Paparone PW. Neuropsychiatric manifestations of Lyme disease. J Am Osteopath Assoc 1998 Jul;98(7):373-8
278. Maes M, Berk M, Goehler L, Song C et al. Depression and sickness behavior are Janus-faced responses to shared inflammatory pathways. BMC Med. 2012; 10: 66. doi: 10.1186/1741-7015-10-66
279. Pasareanu AR, Mygland Å, Kristensen Ø. A woman in her 50s with manic psychosis. Tidsskr Nor Laegeforen. 2012 Mar 6;132(5):537-9.
280. Paterson RW, Takada LT, Geschwind MD. Diagnosis and treatment of rapidly progressive dementias. Neurol Clin Pract. 2012 Sep; 2(3): 187–200. doi: 10.1212/CPJ.0b013e31826b2ae8
281. Petrovic M, Vogelaers D, Van Renterghem L, De Reuck J, Afschrift M. Lyme borreliosis - A review of the late stages and treatment of four cases. Acta Clinica Belgica 1998;53-3:178-183.
282. Pfister HW, Preac-Mursic V, Wilske B, Rieder G, Forderreuther S, Schmidt S, Kapfhammer HP. Catatonic syndrome in acute severe encephalitis due to Borrelia burgdorferi infection. Neurology. 1993 Feb;43(2):433-5.
283. Pfister HW, Tobias A.Rupprecht TA. Clinical aspects of neuroborreliosis and post-Lyme disease syndrome in adult patients. International Journal of Medical Microbiology. 2006 296(1):11-16 https://doi.org/10.1016/j.ijmm.2005.12.003
284. Plutchok JJ, Tikofsky RS, Liegner K, Kochevar JM, Fallon BA, Van Heertum RL. Tc-99m HMPAO Brain SPECT imaging in chronic Lyme disease. J Spiro Tick- borne Dis 1999; 6: 117-122.
285. Plutchok JJ, Tikofsky RS, Liegner KB, Fallon BA, Van Heertum RL. Brain SPECT imaging in chronic Lyme disease. J Spiro Tick Borne-Dis. 1999; 6: 10-16.
286. Pollina DA, Elkins LE, Squires NK, Scheffer SR, Krupp LB. Does process- specific slowing account for cognitive deficits in Lyme disease? Appl Neuropsychol. 1999;6(1):27-32.
287. Pollina DA, Sliwinski M, Squires NK, Krupp LB. Cognitive processing speed in Lyme disease. Neuropsychiatry Neuropsychol Behav Neurol. 1999 Jan;12(1):72-8.
288. Poplawska R, Konarzewska B, Gudel-Trochimowicz I, Szulc A. Psychologic disorders in acute and persistent neuroborreliosis. Pol Merkuriusz Lek 2001 Jan;10(55):36-7
289. Poplawska R, Szulc A, Zajkowska J, Pancewicz S. Neuroborreliosis: a psychiatric problem? Psychiatr Pol 1999 Mar- Apr;33(2):241-50.
290. Preac-Mursic V, Wilske B, Schierz G, et al. Repeated isolation of spirochetes from the cerebrospinal fluid of a patient with meningoradiculitis (Bannwarth syndrome). Eur J Clin Microbiol 1984; 3: 564-565.
291. Primavera A, Gazzola P, De Maria AF. Neuropsychological deficits in neuroborreliosis. Neurology. 1999 Sep 11;53(4):895-6.
292. Puri BK, Shah M, Julu PO, Kingston MC, Monro JA. The association of lyme disease with loss of sexual libido and the role of urinary bladder detrusor dysfunction. Int Neurourol J. 2014 Jun;18(2):95-7.
293. Puri BK et al. The effect of artesunate on short-term memory in Lyme borreliosis. Medical Hypotheses 2017.
294. Putnam FW. Child Development and Dissociation. Child and Adolescent Psychiatric Clinics of North

America. 1996 5(2):285-302. https://doi.org/10.1016/S1056-4993(18)30367-5
295. Quinn SJ, Boucher BJ, Booth JB. Reversible sensorineural hearing loss in Lyme disease. J Laryngol Otol. 1997 Jun;111(6):562-4.
296. Ragnaud JM, Morlat P, Buisson M, Ferrer X, Orgogozo JM, Julien J, Beylot J, Aubertin J. Neurologic manifestations of Lyme disease. Apropos of 25 cases. Rev Med Interne. 1995;16(7):487-94.
297. Ramanan SV. Loss of the sense of humor. Arch Intern Med 2000 Sep 11;160(16):2546 .
298. Ramesh G, Benge S, Pahar B, Philipp MT. A possible role for inflammation in mediating apoptosis of oligodendrocytes as induced by the Lyme disease spirochete Borrelia burgdorferi. J Neuroinflammation. 2012; 9: 72. doi: 10.1186/1742-2094-9-72
299. Ramesh G, Didier PJ, England JD, et al. Inflammation in the pathogenesis of Lyme neuroborreliosis. Am J Pathol. 2015 May;185(5):1344-60.
300. Ramesh R, Borda JT, Dufor J, Kaushal D, Ramamoorthy R, Lackner AA, Philipp MT. Interaction of the Lyme disease spirochete Borrelia burgdorferi with brain parenchyma elicits inflammatory mediators from glial cells as well as glial and neuronal apoptosis. Am J Pathol. 2008; 173:1415-27.
301. Ramesh G, Santana-Gould L, Inglis FM, England JD, Philipp MT. The Lyme disease spirochete Borrelia burgdorferi induces inflammation and apoptosis in cells from dorsal root ganglia. *J Neuroinflammation*. 2013;10:88. Published 2013 Jul 18. doi:10.1186/1742-2094-10-88
302. Ratnasamy N, Everett ED, Roland WE, McDonald G, Caldwell CW. Central nervous system manifestations of human ehrlichiosis. Clin Infect Dis 1996 Aug;23(2):314-9
303. Ravdin LD, Hilton E, Primeau M, Clements C, Barr WB. Memory functioning in Lyme borreliosis. The Journal of Clinical Psychiatry [01 Jul 1996, 57(7):282-286]
304. Rebman AW et al. The clinical symptoms and quality-of-life characterization of a well-defined group of patients with posttreatment Lyme disease syndrome. Front Med (Lausanne) 2017 14:224.
305. Reddy KP, McCannon JB, Venna N. Diaphragm paralysis in Lyme disease: late occurrence in the course of treatment and long-term recovery. Ann Am Thorac Soc. 2015 Apr;12(4):618-20.
306. Reik L, Smith L, Khan A, Nelson W. Demyelinating encephalopathy in Lyme disease. Neurology Feb 1985, 35 (2) 267; DOI: 10.1212/WNL.35.2.267
307. Reik L, Steere AC, Bartenhagen NH, Shope RE, Malawista SE. Neurologic abnormalities of Lyme disease. Medicine (Baltimore). 1979 Jul;58(4):281-94.
308. Rhee H, Cameron DJ. Lyme disease and pediatric autoimmune neuropsychiatric disorders associated with streptococcal infections (PANDAS): an overview. Int J Gen Med. 2012;5: 163-174.
309. Riedel M, Straube A, Schwarz MJ, Wilske B, Muller N. Lyme disease presenting as Tourette's syndrome. Lancet. 1998 Feb 7;351(9100):418-9
310. Roche Lanquetot R, Ader F, Durand MC, Carlier R, Defferriere H, Dinh A, Herrmann JL, Guillemot D, Perronne C, Salomon J. Results of a prospective standardized study of 30 patients with chronic neurological and cognitive disorders after tick bites. Med Mal Infect. 2008 Oct;38(10):543-8.
311. Roelcke U, Barnett W, Wilder-Smith E, Sigmund D, Hacke W. Untreated neuroborreliosis: Bannwarth's syndrome evolving into acute schizophrenia-like psychosis. A case report. J Neurol. 1992 Mar;239(3):129-31
312. Román GC, Sachdev P, Royall DR, Bullock RA et al. Vascular cognitive disorder: a new diagnostic category updating vascular cognitive impairment and vascular dementia. Journal of the Neurological Sciences. 2004 226(1-2):81-7.
313. Rothaus C. A Girl with Seizures SOURCE: NEJM. May 22nd, 2015 http://blogs.nejm.org/now/index.php/a-girl-with-seizures/2015/05/22/comment-page- 1/#comment-225701
314. Rudnik I, Konarzewska B, Zajkowska J, Juchnowicz D, Markowski T, Pancewicz SA. The organic disorders in the course of Lyme disease. Pol Merkuriusz Lek. 2004 Apr;16(94):328-31
315. Rudnik I, Poplawska R, Zajkowska J, Konarzewska B, Juchnowicz D, Pancewicz SA. Mental problems in Lyme disease. Pol Merkuriusz Lek. 2003 Aug;15(86):161-4
316. Rudnik-Szalaj I, Poplawska R, Zajkowska J, Szulc A, Pancewicz SA, Gudel I.Mental disorders in Lyme disease. Pol Merkuriusz Lek. 2001 Nov;11(65):460-2.
317. Rundell JR, Wise MG. Neurosyphilis: a psychiatric perspective. Psychosomatics. 1985; 26: 287-295.
318. Samuel B, Axelband J, Mckim K, Leh D. Borrelia burgdorferi: A clinical chameleon. Consultant. 2015;55(7):530-535.
319. Sanders K, Rogers JD. Lyme encephalopathy. Neurology. 1991 Jun;41(6):952- 3.
320. Savely VR, Stricker RB. Morgellons disease: Analysis of a population with clinically confirmed microscopic

subcutaneous fibers of unknown etiology. Clin Cosmet Investig Dermatol. 2010; 3: 67–78.
321. Savely VR. Update on Lyme disease: the hidden epidemic. J Infus Nurs. 2008 Jul-Aug;31(4):236-40.
322. Schaller JL, Burkland GA, Langhoff PJ. Do bartonella infections cause agitation, panic disorder, and treatment-resistant depression? MedGenMed. 2007 Sep 13;9(3):54.
323. Scerpella TA, Engber WD. Chronic Lyme disease arthritis: review of the literature and report of a case of wrist arthritis. J Hand Surg Am. 1992 May;17(3):571-5.
324. Scheffer RE, Linden S. Concurrent medical conditions with pediatric bipolar disorder. Curr Opin Psychiatry. 2007 Jul;20(4):398-401. Review.
325. Schmidt H, Djukic M, Jung K, Holzgraefe M, Dechent P, von Steinbüchel N, Blocher J, Eiffert H, Schmidt-Samoa C. Neurocognitive functions and brain atrophy after proven neuroborreliosis: a case-control study. BMC Neurol [2015]
326. Schneider RK, Robinson MJ, Levenson JL Psychiatric presentations of non-HIV infectious diseases. Psychiatr Clin North Am 2002 Mar;25(1):1-16
327. Schoof J, Kluge C, Heinze HJ, Galazky I.Startle myoclonus induced by Lyme neuroborreliosis: a case report. J Med Case Rep 2013(May); 7(1): 124. DOI: 10.1186/1752-1947-7-124
328. Schuler PA. Gifted Students and Lyme Disease: What Educators, Counselors, and Parents Need to Know. Gifted Child Today 2013 36: 35. DOI:10.1177/1076217512465288
329. Seltzer EG, Gerber MA, Cartter ML, Freudigman K, Shapiro ED. Long-term Outcomes of Persons with Lyme Disease. *JAMA.* 2000;283(5):609–616. doi:10.1001/jama.283.5.609
330. Shadick NA, Phillips CB, Logigian EL, Steere AC, Kaplan RF, Berardi VP, Duray PH, Larson MG, Wright EA, Ginsburg KS, Katz JN, Liang MH. The long-term clinical outcomes of Lyme disease. A population-based retrospective cohort study. Ann Intern Med. 1994 Oct 15;121(8):560-7.
331. Shamim A, Shamim S; Liss G; Nylen E; Pincus J; Yepes M. Constipation Heralding Neuroborreliosis Arch Neurol. 2005;62:671-673.
332. Sherr VT. "Bell's Palsy of the Gut" and Other GI Manifestations of Lyme and Associated Diseases A SPECIAL ARTICLE. Practical Gastroenterology. 2006/04/01 30.
333. Sherr VT. Human babesiosis--an unrecorded reality. Med Hypotheses. 2004;63(4):609-15
334. Sherr VT. Munchausen's syndrome by proxy and Lyme disease: medical misogyny or diagnostic mystery? Med Hypotheses. 2005;65(3):440-7.
335. Sherr VT. Panic attacks may reveal previously unsuspected chronic disseminated Lyme disease. J Psychiatr Pract. 2000 Nov;6(6):352-6.
336. Shotland LI, Mastrioanni MA, Choo DL, Szymko-Bennett YM, Dally LG, Pikus AT, Sledjeski K, Marques A. Audiologic manifestations of patients with post-treatment Lyme disease syndrome. Ear Hear. 2003 Dec;24(6):508-17
337. Shprecher D, Kurlan R. The Management of Tics. Mov Disord. Author manuscript; available in PMC 2009 Jun 24.Published in final edited form as: Mov Disord. 2009 Jan 15; 24(1): 15–24. doi: 10.1002/mds.22378
338. Shulman KI. Disinhibition syndromes, secondary mania and bipolar disorder in old age. Journal of Affective Disorders. 1997, 46(3):175-182 https://doi.org/10.1016/S0165-0327(97)00156-0.
339. Smith AJ, Oertle J, Prato D. Borrelia burgdorferi: Cell biology and clinical manifestations in latent chronic Lyme. Open J Med Microbiol. 2014;4:210-223.
340. Smith IS, Rechlin DP. Delayed diagnosis of neuroborreliosis presenting as bell palsy and meningitis. J Am Osteopath Assoc. 2010 Aug;110(8):441-4.
341. Smith V, Traquina DN. Pediatric bilateral facial paralysis. Laryngoscope. 1998 Apr;108(4 Pt 1):519-23.
342. Sno HN. Signs and significance of a tick-bite: psychiatric disorders associated with Lyme disease. Tijdschr Psychiatr. 2012;54(3):235-43.
343. Sparsa L, Blanc F, Lauer V, Cretin B, Marescaux C, Wolff V. Recurrent ischemic strokes revealing Lyme meningovasculitis. Rev Neurol (Paris). 2009 Mar;165(3):273-7.
344. Staci D. BilboSD, Jaclyn M. Schwarz JM. Early-life programming of later-life brain and behavior: a critical role for the immune system. Frontiers in Behvrioral Neurosciences. 2009:3
345. Steere AC. A 58-year-old man with a diagnosis of chronic Lyme disease, 1 year later. JAMA. 2002 Aug 28;288(8):1002- 10.
346. Stein SL, Solvason HB, Biggart E, Spiegel D. A 25-year-old woman with hallucinations, hypersexuality, nightmares, and a rash. Am J Psychiatry. 1996 Apr;153(4):545-51.
347. Stevens JR, Prince JB, Prager LM, Stern TA. Psychotic disorders in children and adolescents: a primer on contemporary evaluation and management. *Prim Care Companion CNS Disord*. 2014;16(2):PCC.13f01514.

doi:10.4088/PCC.13f01514
348. Stewart T. Common dementing illnesses associated with prominent and disturbing behavioral changes. Geriatrics . 2006, 61(8):23-27.
349. Stratmoen M.Neurological complications of Lyme disease: Dilemmas in Diagnosis and Treatment. Neurology Today 2004;4(4)71-5.
350. Stricker RB, Winger EE. Holmes-Adie syndrome and Lyme disease. Lancet. 2001 Mar 10;357(9258):805.
351. Stricker RB, Johnson L (2014) Lyme Disease: Call for a "Manhattan Project" to Combat the Epidemic. PLoS Pathog 10(1): e1003796. https://doi.org/10.1371/journal.ppat.1003796
352. Stricker RB, Winger EE. Musical hallucinations in patients with Lyme disease. South Med J 2003; 96(7):711-715 .
353. Stricker RB, Green CL, Savely VR, Chamallas SN, Johnson L. Safety of intravenous antibiotic therapy in patients referred for treatment of neurologic Lyme disease. Minerva Med. 2010 Feb;101(1):1-7.
354. Stricker RB, DeLong AK, Green CL, Savely VR, Chamallas SN, Johnson L. Benefit of intravenous antibiotic therapy in patients referred for treatment of neurologic Lyme disease. Int J Gen Med 2011; 4: 639-646.
355. Stricker RB, Johnson L. Anti-neural antibody reactivity in patients with a history of Lyme borreliosis and persistent symptoms. Brain Behavior Immun. 2010;24: 1025 .
356. Sumiya H, Kobayashi K, Mizukoshi C, Aoki T, Koshino Y, Taki J, Tonami N. Brain perfusion SPECT in Lyme neuroborreliosis. J Nucl Med. 1997 Jul;38(7):1120-2.
357. Svetina C, Barr WB, Rastogi R, Hilton E. The neuropsychological examination of naming in Lyme borreliosis. Appl Neuropsychol. 1999;6(1):33-8.
358. Tager FA, Fallon BA, Keilp J, Rissenberg M, Jones CR, Liebowitz MR. A controlled study of cognitive deficits in children with chronic Lyme disease. J Neuropsychiatr Clin Neurosci 2001;13:500-507.
359. Tager FA, Fallon BA. Psychiatric and cognitive features of Lyme disease. Psychiatr Ann 2001; 31: 173-181.
360. Touradji P, Aucott JN, Yang T, Rebman AW, Bechtold KT. Cognitive Decline in Post-treatment Lyme Disease Syndrome. Arch Clin Neuropsychol [2019]
361. Treib J, Grauer MT, Haass A, Langenbach J, Holzer G, Woessner R. Chronic fatigue syndrome in patients with Lyme borreliosis. Eur Neurol. 2000;43(2):107-9.
362. Tselis A, MD, Booss J. Behavioral consequences of infections of the central nervous system: with emphasis on viral infections. J Am Acad Psychiatry Law 2003;31:289–98.
363. Tucci V, Moukaddam N, Meadows J et al. The Forgotten Plague: Psychiatric Manifestations of Ebola, Zika, and Emerging Infectious Diseases. J Glob Infect Dis. 2017 Oct-Dec; 9(4): 151–156. doi: 10.4103/jgid.jgid_66_17
364. Vamos E, Pardutz A, Klivenyi P, Toldi J, Vecsei L The role of kynurenines in disorders of the central nervous system: Possibilities for neuroprotection. J Neurol Sci. 2009 Mar 4.
365. van den Bergen HA, Smith JP, van der Zwan A. Lyme Psychosis. Ned Tijdschr Geneeskd 1993 Oct 9;137(41):2098-100.
366. Vázquez M, Sparrow SS, and Shapiro ED. Long-term neuropsychologic and health outcomes of children with facial nerve palsy attributable to Lyme disease. Pediatrics 2003; 112(2): e93-e97.
367. Venkatesan A, Williams MA. Case 13: A Man with Progressive Headache and Confusion. MedGenMed. 2006; 8(3): 19. Published online 2006 Jul 26.
368. Vital C, Vital A, Lagueny A, Larribau E, Saintarailles J, Julien J. Subacute inflammatory polyneuropathy: two cases with plasmacytoid histiocytes in the endoneurium. Ultrastruct Pathol. 1998 Sep-Oct;22(5):377-83.
369. Volkman D Anti-neural antibody reactivity in patients with a history of Lyme borreliosis and persistent symptoms. Brain Behavior Immun. 2010;24:1026.
370. Waniek C, Prohovnik I, Kaufman MA, Dwork AJ. Rapidly progressive frontal- type dementia associated with Lyme disease. J Neuropsychiatry Clin Neurosci 1995;7(3):345-7.
371. Warneke L. Managing resistant depression. When patients do not respond to therapy. *Can Fam Physician*. 1993;39:843–850.
372. Weder B, Wiedersheim P, Matter L, Steck A, Otto F. Chronic progressive neurological involvement in Borrelia burgdorferi infection. J Neurology 1987;234:40-43.
373. Weissenbacher S, Ring J, Hofmann H. Gabapentin for the symptomatic treatment of chronic neuropathic pain in patients with late-stage Lyme borreliosis: a pilot study. Dermatology. 2005;211(2):123-7.
374. Westervel HJ, McCaffrey RJ. Neuropsychological functioning in chronic Lyme disease. Neuropsychol Rev 2002 Sep;12(3):153-77 Review.
375. Wilke M, Eiffert H, Christen HJ, Hanefeld F. Primarily chronic and cerebrovascular course of Lyme

neuroborreliosis: case reports and literature review. Arch Dis Child. 2000 Jul;83(1):67-71. Review.
376. Wills AB, Spaulding AB, Adjemian J, et al. Long-term Follow-up of Patients with Lyme Disease: Longitudinal Analysis of Clinical and Quality-of-life Measures, *Clinical Infectious Diseases*. PlumX Metrics. 2016, 62(12): 1546–1551. DOI: https://doi.org/10.1016/0738-081X(93)90095-T
377. Woessner R, Treib J. Pain, fatigue, depression after borreliosis. Antibiotics used up--what next? MMW Fortschr Med. 2003 Sep 18;145(38):45-8.
378. Wokke JHJ, van Gijn J, Elderson A, Stanek G. Chronic forms of Borrelia burgdorferi infection of the nervous system. Neurology 1987;37:1031-1034.
379. Wurtz R, Psychiatric Diseases Presenting as Infectious Diseases, *Clinical Infectious Diseases*. 1998, 26(4): 924–932, https://doi.org/10.1086/513936
380. Mendez, M.F. Mania in Neurologic Disorders. Curr Psychiatry Rep (2000) 2: 440. https://doi.org/10.1007/s11920-000-0030-6
381. Yolken RH, Torrey EF. Are some cases of psychosis caused by microbial agents? A review of the evidence. Mol Psychiatry. 2008 May;13(5):470-9.
382. Younger DS, Rosoklija G, Hays AP. Persistent painful Lyme radiculoneuritis. Muscle Nerve. 1995 Mar;18(3):359-60.
383. Younger DS. Serial Brain Positron Emission Tomography Fused to Magnetic Resonance Imaging in Post-Infectious and Autoantibody-Associated Autoimmune Encephalitis. World Journal of Neuroscience. 2019, 9, 153-156. DOI: 10.4236/wjns.2019.93010 http://www.scirp.org/Journal/paperinformation.aspx?paperid=94154
384. Young JL. Chronic Lyme disease linked to ADHD in adults. American Psychiatric Association 2012 Annual Meeting. Medscape. May 2 2012. http://www.medscape.com/viewarticle/763458
385. Zajkowska JM, Hermanowska-Szpakowicz T, Kondrusik M, Pancewicz SA. Neurologic syndromes in Lyme disease. Pol Merkuriusz Lek. 2000 Aug;9(50):584-8. Review.
386. Zajkowska JM, Poplawska R, Pancewicz SA, Kondrusik M, Gudel I, Snarska I. Mental disorders in the course of neuroborreliosis: own observation. Psychiatr Pol 1999 Nov-Dec;33(6):939-46.
387. Zamponi N, Cardinali C, Tavoni MA, Porfiri L, Rossi R, Manca A. Chronic neuroborreliosis in infancy. Ital J Neurol Sci. 1999; 20:303-307.
388. Zhang Y, Lafontant G, Bonner FJ Jr. Lyme neuroborreliosis mimics stroke: a case report. Arch Phys Med Rehabil. 2000 Apr;81(4):519-21.

Tick-Borne Diseases and Dementia

1. Aboul-Enein F, Kristoferitsch W. Normal pressure hydrocephalus or neuroborreliosis? Wien Med Wochenschr. 2009;159(1-2):58-61.
2. Annals of the New York Academy of Sciences, Lyme Disease and Related Disorders. 539, 468–470.
3. Bannwarth, A. Zur Klinik und Pathogenese der chronischen lymphocytären Meningitis. Arch Psychiatr Nervenkr. 1944;117: 161- 185.
4. Blanc F, Philippi N, Cretin B, Kleitz C, Berly L, Jung B, Kremer S, Namer IJ, Sellal F, Jaulhac B, de Seze J. Lyme neuroborreliosis and dementia. J Alzheimers Dis. 2014; 41(4): 1087-93.
5. Duyckaerts C, Delatour B, Potier MC. Classification and basic pathology of Alzheimer disease. Acta Neuropathol 2009;118, 5-36
6. Galbussera A, Tremolizzo L, Isella V, et al. Lack of evidence for Borrelia burgdorferi seropositivity in Alzheimer disease. Alzheimer Dis Assoc Disord 2008;22(3), 308.
7. Guo JP, Arai T, Miklossy J, McGeer PL. Aβ and tau form soluble complexes that may promote self-aggregation of both into the insoluble forms observed in Alzheimer’s disease. Proc Natl Acad Sci U S A. 2006 Feb 7;103(6):1953-8.
8. Haass C, Selkoe DJ. Soluble protein oligomers in neurodegeneration: lessons from the Alzheimer's amyloid beta-peptide. Nat Rev Mol Cell Biol 2007;8: 101-12.
9. Hardy J, Selkoe DJ. The amyloid hypothesis of Alzheimer's disease: progress and problems on the road to therapeutics. Science 2002;297: 353-6
10. Holmes C, Cotterell D. Role of infection in the pathogenesis of Alzheimer's disease: implications for treatment. CNS Drugs 2009;23(12): 993-1002.

11. Honjo K, van Reekum R, Verhoeff NP. Alzheimer's disease and infection: Do infectious agents contribute to progression of Alzheimer's disease? Alzheimers Dement. 2009;5: 348-360 .
12. Itzhaki RF, Lathe R, Balin BJ, Ball MJ, Bearer EL, Braak H, Bullido MJ, Carter C, Clerici M, Cosby SL, Del Tredici K, Field H, Fulop T, Grassi C, Griffin WS, Haas J, Hudson AP, Kamer AR, Kell DB, Licastro F, Letenneur L, Lövheim H, Mancuso R, Miklossy J, Otth C, Palamara AT, Perry G, Preston C, Pretorius E, Strandberg T, Tabet N, Taylor-Robinson SD, Whittum-Hudson JA. Microbes and Alzheimer's Disease. J Alzheimers Dis. 2016;51(4):979-84.
13. Jack CR Jr, Knopman DS, Jagust WJ, Shaw LM, Aisen PS, Weiner MW, Petersen RC, Trojanowski JQ. Hypothetical model of dynamic biomarkers of the Alzheimer's pathological cascade. Lancet Neurol 2010;9:119-28.
14. Khan UA, Liu L, Provenzano FA, Berman DE, Profaci CP, Sloan R, Mayeux R, Duff KE, Small SA. Molecular drivers and cortical spread of lateral entorhinal cortex dysfunction in preclinical Alzheimer's disease. Nat Neurosci. 2014 Feb;17(2):304-11.
15. Lue LF, Kuo YM, Roher AE, Brachova L, Shen Y, Sue L, Beach T, Kurth JH, Rydel RE, Rogers J. Soluble amyloid beta peptide concentration as a predictor of synaptic change in Alzheimer's disease. Am J Pathol 1999;155: 853-862
16. MacDonald AB. Concurrent neocortical Borreliosis and Alzheimer's disease: Demonstration of a spirochetal cyst form. Ann NY Acad Sci 1988;539:468-470.
17. MacDonald AB, Miranda JM. Concurrent neocortical borreliosis and Alzheimer's disease. Human Pathol 1987; 18: 759-761.
18. MacDonald AB. Concurrent neocortical borreliosis and Alzheimer's disease: demonstration of a spirochetal cyst form. Ann NY Acad Sci 1988; 539: 468-470.
19. MacDonald AB, Berger BW, and Schwan TG. Clinical implications of delayed growth of the Lyme disease spirochete, Borrelia burgdorferi. Acta Trop 1990 48; (2): 89-94.
20. MacDonad AB. In situ DNA hybridization study of granulovacuolar degeneration in human Alzheimer autopsy neurons for flagellin b transcriptomes of Borrelia burgdorferi. Alzheimer's Dis Dementia 2006; 2 (Suppl. 1): S207.
21. MacDonald AB. Cystic borrelia in Alzheimer's disease and in non-dementia neuroborreliosis. Alzheimer's Dementia 2006; 2 (Suppl. 1):S433.
22. MacDonald AB. Plaques of Alzheimer's disease originate from cysts of Borrelia burgdorferi, the Lyme disease spirochete. Med Hypotheses. 2006;67(3):592-600.
23. MacDonald AB. Spirochetal cyst forms in neurodegenerative disorders...hiding in plain sight. Med Hypotheses. 2006;67(4):819-32. Epub 2006 Jul 7.
24. MacDonald AB. Transfection "Junk" DNA - a link to the pathogenesis of Alzheimer's disease? Med Hypotheses. 2006;66(6):1140-1.
25. MacDonald AB. Alzheimer's neuroborreliosis with trans-synaptic spread of infection and neurofibrillary tangles derived from intraneuronal spirochetes. Med Hypotheses 2007; 68: 822-825. [7 of 10 cases of Alzheimer's disease had B. burgdorferi in their brains].
26. MacDonald AB. Alzheimer's disease Braak Stage progressions reexamined and redefined as Borrelia infection transmission through neural circuits. Med Hypotheses. 2007; 68(5): 1059-64
27. MacDonald AB, Miranda JM. Concurrent neocortical borreliosis and Alzheimer's disease. Hum Pathol. 1987 Jul;18(7):759-61.
28. MacDonald AB. Plaques of Alzheimer's disease originate from cysts of Borrelia burgdorferi, the Lyme disease spirochete. Med Hypotheses. 2006;67(3):592-600.
29. Macdonald AB. Transfection ''junk'' DNA – A link to the pathogenesis of Alzheimer's disease? Medical Hypothesis. 2006;66(6):1140-1.
30. MacDonald AB. Borrelia in the brains of patients dying with dementia. JAMA. 1986;256:2195-2196.
31. MacDonald AB. Alzheimer's neuroborreliosis with trans-synaptic spread of infection and neurofibrillary tangles derived from intraneuronal spirochetes. Med Hypotheses. 2007;68(4):822-5. Epub 2006 Oct 20.
32. MacDonald AB. Concurrent neocortical borreliosis and Alzheimer's disease: Demonstration of a spirochetal cyst form. Ann N Y Acad Sci 1988; 539:468-470.
33. McLaughlin R, Kin NM, Chen MF, et al. Alzheimer's disease may not be a spirochetosis. Neuroreport 1999;10(7), 1489-91.
34. Meer-Scherrer L, Chang Loa C, Adelson ME, Mordechai E, Lobrinus JA, Fallon BA, Tilton RC. Lyme disease associated with Alzheimer's disease. Curr Microbiol. 2006 Apr;52(4):330-2.

35. Miklossy J. Alzheimer's disease — a spirochetosis? NeuroReport 1993; 4: 841-848.
36. Miklossy J, Gern L, Darekar P, Janzer RC, Loos H. Senile plaques, neurofibrillary tangles and neuropil threads contain DNA? J Spiro Tick-borne Dis 1995; 2: 1-5.
37. Miklossy JM, Khalili K, Gern L, Ericson RL, Darekar P, Bolle L, Hurlimann J, and Paster BJ. Borrelia burgdorferi persists in the brain in chronic Lyme neuroborreliosis and may be associated with Alzheimer's disease. J Alzheimers Dis 2004; 6; 639-649.
38. Miklossy J. Chronic inflammation and amyloidogenesis in Alzheimer's disease — role of spirochetes. J Alzheimers Dis 2008; 13: 381-391.
39. Miklossy J, Kasas S, Zurn AD, McCall S, Yu S, and McGeer PL. Persisting atypical and cystic forms of Borrelia burgdorferi and local inflammation in Lyme neuroborreliosis. J Neuroinflammation 2008; 5: 40-57.
40. Miklossy J. Chronic or late Lyme neuroborreliosis: analysis of evidence compared to chronic or late neurosyphilis. Open Neurol J 2012;6: 146-157.
41. Miklossy J. Chronic inflammation and amyloidogenesis in Alzheimer's disease -- role of spirochetes. Alzheimers Dis. 2008 May;13(4):381-91. Review.
42. Miklossy J. Alzheimer's disease - a neurospirochetosis. Analysis of the evidence following Koch's and Hill's criteria. J Neuroinflammation. 2011 Aug 4;8(1):90.
43. Miklossy J. Historic evidence to support a causal relationship between spirochetal infections and Alzheimer's disease. Frontiers Aging Neurosci. 2015 Apr 16;7:46.
44. Miklossy J. Bacterial amyloid and DNA are important constituents of senile plaques: Further evidence of the spirochetal and biofilm nature of senile plaques. J Alzheimer's Dis. 2016;53:1459-1473.
45. Miklossy J. Emerging roles of pathogens in Alzheimer disease. Expert Rev Mol Med. 2011 Sep 20;13:e30.
46. Miklossy J, Donta SE, Mueller K, Nolte O, Perry G. Chronic or late Lyme neuroborreliosis: present and future. Open Neurol J. 2012;6:78.
47. Miklossy J, Kasas S, Janzer RC, Ardizzoni F, Van der Loos H. Further ultrastructural evidence that spirochaetes may play a role in the aetiology of Alzheimer's disease. Neuroreport. 1994 Jun 2;5(10):1201-4.
48. Miklossy J, Kasas S, Zurn AD, McCall S, Yu S, McGeer PL. Persisting atypical and cystic forms of Borrelia burgdorferi and local inflammation in Lyme neuroborreliosis. J. Neuroinflammation. 2008;5: 40.
49. Miklossy J, Khalili K, Gern L, Ericson RL, Darekar P, Bolle L, Hurlimann J, Paster BJ. Borrelia burgdorferi persists in the brain in chronic Lyme neuroborreliosis and may be associated with Alzheimer disease. J Alzheimer's Disease 2004;6 (6): 639-49; discussion 673-681.
50. Miklossy J, Kris A, Radenovic A, Miller L, Forro L, Martins R, Reiss K, Darbinian N, Darekar P, Mihaly L, Khalili K. Beta-amyloid deposition and Alzheimer's type changes induced by Borrelia spirochetes. Neurobiol Aging. 2006 Feb;27(2):228-36.
51. Miklossy J, Kuntzer T, Bogousslavsky J, Regli F, Janzer RC. Meningovascular form of neuroborreliosis: Similarities between neuropathological findings in a case of Lyme disease and those occurring in tertiary neurosyphilis. Acta Neuropathol 1990;80. 568- 572.
52. Miklossy J, Taddei K, Martins R et al. Alzheimer disease: curly fibers and tangles in organs other than brain. J Neuropathol Exp Neurol. 1999;58, 803-814.
53. Miklossy J, Van der Loos H. (1991) The long-distance effects of brain lesions: A study of myelinated pathways in the human brain using polarizing and fluorescence microscopy. J Neuropathol Exp Neurol 50, 1-15.
54. Miklossy J. (2012) Chronic or late lyme neuroborreliosis: analysis of evidence compared to chronic or late neurosyphilis. Open Neurol J. 6, 146-57.
55. Miklossy J. Alzheimer's disease - a neurospirochetosis. Analysis of the evidence following Koch's and Hill's criteria. J Neuroinflammation. 2011 Aug 4;8(1):90.
56. Miklossy J. Biology and neuropathology of dementia in syphilis and Lyme disease. Handb Clin Neurol. 2008;89:825-44.
57. Miklossy J. Chronic inflammation and amyloidogenesis in Alzheimer's disease -- role of spirochetes. J Alzheimers Dis. 2008 May;13(4):381-91.
58. Miklossy J. Historic evidence to support a causal relationship between spirochetal infections and Alzheimer's disease. Frontiers in Aging Neuroscience. 2015 Apr 16;7:46.
59. Miklossy J. Alzheimer's disease--a spirochetosis? Neuroreport. 1993 Jul;4(7):841-8.
60. Miklossy J. The lack of correlation between the incidence of Lyme disease and deaths due to Alzheimer's disease cannot reflect the lack of involvement of Borrelia burgdorferi in Alzheimer's dementia. J Alzheimers Dis 2014;42:115-118.

61. Nilsson P et al. Aβ secretion and plaque formation depend on autophagy. Cell Reports 2013;5(1): 61-69.
62. Neugroschl J, Wang S. Alzheimer's Disease: Diagnosis and Treatment Across the Spectrum of Disease Severity. Mt Sinai J Med. Author manuscript; available in PMC 2012 Mar 29.Published in final edited form as: Mt Sinai J Med. 2011 Jul-Aug; 78(4): 596–612. doi: 10.1002/msj.20279
63. Almeida OP, Nicola T. Lautenschlager. Dementia associated with infectious diseases. International Psychogeriatrics. 2005; 17(Suppl): S65–S77.
64. Pappolla MA, Omar R, Saran B, et al. Concurrent neuroborreliosis and Alzheimer's disease: analysis of the evidence. Hum Pathol 1989;20(8): 753-7.
65. Riek R. Infectious Alzheimer's disease? Nature 2006;444: 429-431 .
66. Ruitenberg A, den Heijer T, Bakker SL, van Swieten JC, Koudstaal PJ, Hofman A, Breteler MM. Cerebral hypoperfusion and clinical onset of dementia: the Rotterdam study. Ann Neurol. 2005;57: 789-94.
67. Selkoe DJ. Preventing Alzheimer's disease. Science 2012;337: 1488-92.
68. Suter O-C, Sunthorn T, Kraftsik R, Straubel J, Darekar P, Khalili K, Miklossy J. Cerebral Hypoperfusion generates cortical watershed microinfarcts in Alzheimer disease. Stroke 2002;33: 1986-1992 .
69. Verdile G, Gnjec A, Miklossy J, Fonte J, Veurink G, Bates K, Kakulas B, Mehta PD, Milward EA, Tan N, Lareu R, Lim D, Dharmarajan A, Martins RN. Protein markers for Alzheimer disease in the frontal cortex and cerebellum. Neurology. 2004 Oct 26;63(8):1385-92.
70. Waniek C, Prohovnik I, Kaufman MA, Dwork AJ. Rapidly progressive frontal-type dementia associated with Lyme disease. J Neuropsych Clin Neurosci. 1995;7(3):345-7.
71. Williams WM, Torres S, Siedlak SL, Castellani RJ, Perry G, Smith MA, Zhu X. Antimicrobial peptide beta-defensin-1 expression is upregulated in Alzheimer's brain. J Neuroinflammation. 2013;10(1): 127.
72. Zajkowska JM, Hermanowska-Szpakowicz T. New aspects of the pathogenesis of Lyme disease. Przegl Epidemiol 2002;56 (Suppl 1):57-67.
73. Zlokovic BV. Neurovascular pathways to neurodegeneration in Alzheimer's disease and other disorders. Nat Rev Neurosci 2011;12, 723-38.
